# Three chromosome-level duck genome assemblies provide insights into genomic variation during domestication

**DOI:** 10.1038/s41467-021-26272-1

**Published:** 2021-10-11

**Authors:** Feng Zhu, Zhong-Tao Yin, Zheng Wang, Jacqueline Smith, Fan Zhang, Fergal Martin, Denye Ogeh, Maxwell Hincke, Fang-Bing Lin, David W. Burt, Zheng-Kui Zhou, Shui-Sheng Hou, Qiang-Sen Zhao, Xiao-Qin Li, Si-Ran Ding, Guan-Sheng Li, Fang-Xi Yang, Jing-Pin Hao, Ziding Zhang, Li-Zhi Lu, Ning Yang, Zhuo-Cheng Hou

**Affiliations:** 1 National Engineering Laboratory for Animal Breeding and Key Laboratory of Animal Genetics, Breeding and Reproduction, MARA; College of Animal Science and Technology, China Agricultural University, No. 2 Yuanmingyuan West Rd, Beijing, 100193 China; 2 The Roslin Institute & R(D)SVS, University of Edinburgh, Easter Bush, Midlothian, EH25 9RG UK; 3 European Molecular Biology Laboratory, European Bioinformatics Institute, Wellcome Genome Campus, Hinxton, Cambridge, CB10 1SD UK; 4 Department of Cellular and Molecular Medicine, Department of Innovation in Medical Education, Faculty of Medicine, University of Ottawa, 451 Smyth Road, Ottawa, KIH 8M5 Canada; 5 The University of Queensland, St. Lucia, QLD 4072 Australia; 6 Key Laboratory of Animal (Poultry) Genetics Breeding and Reproduction, Ministry of Agriculture and Rural Affairs; State Key Laboratory of Animal Nutrition, Institute of Animal Science, Chinese Academy of Agricultural Sciences, No. 2 Yuanmingyuan West Rd, Beijing, 100193 China; 7 Beijing Golden-Star Inc., Beijing, 100076 China; 8 State Key Laboratory of Agrobiotechnology, College of Biological Sciences, China Agricultural University, Beijing, 100193 China; 9 Institute of Animal Husbandry and Veterinary Science, Zhejiang Academy of Agricultural Sciences, Hangzhou, 310021 China

**Keywords:** Agricultural genetics, Genomics

## Abstract

Domestic ducks are raised for meat, eggs and feather down, and almost all varieties are descended from the Mallard (Anas platyrhynchos). Here, we report chromosome-level high-quality genome assemblies for meat and laying duck breeds, and the Mallard. Our new genomic databases contain annotations for thousands of new protein-coding genes and recover a major percentage of the presumed “missing genes” in birds. We obtain the entire genomic sequences for the C-type lectin (CTL) family members that regulate eggshell biomineralization. Our population and comparative genomics analyses provide more than 36 million sequence variants between duck populations. Furthermore, a mutant cell line allows confirmation of the predicted anti-adipogenic function of NR2F2 in the duck, and uncovered mutations specific to Pekin duck that potentially affect adipose deposition. Our study provides insights into avian evolution and the genetics of oviparity, and will be a rich resource for the future genetic improvement of commercial traits in the duck.

## Abstract (web summary)

Domestic ducks are descended from the Mallard and have been bred for several purposes. Here the authors present three high-quality duck genome assemblies which recover previously missing genes and provide a rich resource of variants potentially related to domestication.

## Introduction

The duck (*Anas platyrhynchos*) is a major source of meat and eggs for human consumption and is also a significant source of feather down. Recent studies have shown that most domestic duck breeds originated from the Mallard about 2200–2500 years ago^1,2^. The Mallard has been confirmed as a major reservoir for avian influenza A viruses^3–5^, thus making it an important model for zoonotic disease studies. The domestic duck represents an excellent model for dissecting genetic mechanisms underlying domestication, due to its short generation interval, high reproduction ability, and extensive history of artificial selection^1,2^. Intensive artificial selection has resulted in very diverse phenotypes within and between domestic ducks compared with Mallard, due to diversification of body size, reproduction, and plumage color, which has generated two major breed types: laying and meat ducks^2^. The Pekin duck (a meat-type breed) is a world standard breed and is famous for its fast growth rate and superior adipose deposition; on the other hand, the Shaoxing (a laying-type breed) duck is recognized for its reproduction ability and has been widely used in laying duck breeding.

High-quality reference genomes of domesticated breeds and their wild relatives are critically important for understanding the genetic basis of phenotype differences. Alterations in genome size and organization, as well as large structural variations (SV), have been observed between the wild ancestor and domesticated descendants in chicken^6,7^ and pig^8^ breeds. The current duck reference genomes (BGI 1.0 and CAU1.0) are derived from the Pekin duck^3^, and still require further improvement in assembly quality metrics, for example with regard to fragment sizes and the number of gaps.

All birds, most reptiles, and formerly dinosaurs lay calcareous eggs, which is a successful reproductive adaptation to a desiccating terrestrial environment^9,10^. The avian egg represents the most advanced amniotic egg in oviparous vertebrates^9^. Understanding the genetics of eggshell biomineralization would be a crucial step in our understanding of the evolution of these unique eggshell features. However, there are still few well-assembled avian genomes, which hinders progress in understanding the relationship between genetics and evolution.

Here, we have produced very high-quality genome assemblies of Mallard, Pekin duck, and Shaoxing duck. These assemblies have uncovered genes previously presumed “missing” in birds. For example, we have obtained the entire genomic sequences for the C-type lectin (CTL) family members that regulate eggshell biomineralization. Finally, our work has identified 36.8 million whole-genome level variations (SNP, indels, structural and chromosomal) among wild and domesticated duck populations, and demonstrated the anti-adipogenic function of *NR2F2* and the changes responsible for differences in adipose deposition between meat-type breeds (such as the Pekin Duck) and its wild ancestor, the Mallard.

## Results

### Genome assembly and annotation

Three chromosome-level assemblies for Mallard (CAU_wild_1.0), laying-type duck (Shaoxing duck, CAU_Laying_1.0), and meat-type duck (Pekin duck, CAU_Pekin_2.0) were built using a multi-level approach, including four different sequencing and assembly technologies (single-molecule real-time sequencing, PacBio SMRT; BioNano optical mapping; high-throughput chromosome conformation capture techniques, Hi–C; Illumina HiSeq) (Supplementary Fig. 1). A total of 112–114 Gb of PacBio long reads were collected for each duck genome, with approximately 100-fold high-quality sequence coverage for each assembly. A summary of statistics for these genome assemblies and total raw reads are shown in Supplementary Tables 1–6. The contig N50 lengths are 5.46 Mb (Pekin duck), 3.79 Mb (Shaoxing duck), and 4.68 Mb (Mallard). Final scaffold N50 lengths are 76.28 Mb (Pekin duck), 76.92 Mb (Shaoxing duck), and 77.63 Mb (Mallard). The final assembled genome sizes are 1.19 Gb (Pekin duck), 1.21 Gb (Shaoxing duck), and 1.21 Gb (Mallard). After clustering of high-throughput chromosome conformation capture (Hi-C) data, the largest 40 super-scaffolds (CAU_wild_1.0: 41) appear to represent chromosomes consisting of 94.62% (Mallard), 95.52% (Shaoxing duck), and 95.26% (Pekin duck) of all sequences, respectively (Supplementary Figs. 2–6, Supplementary Tables 5 and 6). After genome annotation (see Methods), we obtained 18,490, 18,723, and 18,507 annotated protein-coding genes for Mallard, Shaoxing, and Pekin duck genomes, respectively (Supplementary Tables 7 and 8). We also predicted 1270 (Mallard), 1817 (Shaoxing duck), and 1654 (Pekin duck) noncoding RNA genes (Supplementary Tables 9–11). Moreover, we observed that repetitive element (TE) sequences make up ~17% of the total assembly of each duck genome, with long terminal repeat retrotransposons (LTR-RTs) being the most abundant (~13%) (Supplementary Table 12).

**Fig. 1:**
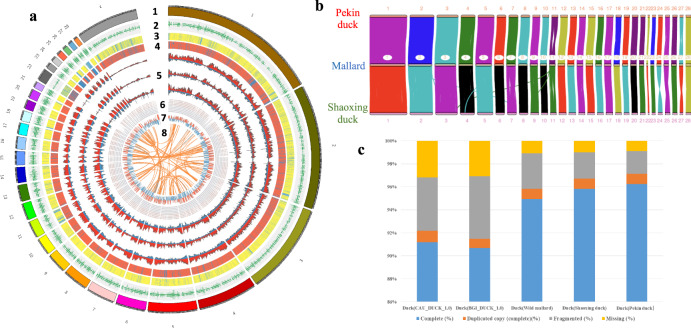
Overview of the assembly quality and characteristics of the duck genome. **a** Chromosomal features of three duck genomes with the integration of genetics (from Chr1 to Chr25). 1: Chromosomal length of Mallard genome (Mb); 2: Gene density (100 kb window); 3: Genome collinearity of Shaoxing duck to Mallard, yellow represents the same orientation, blue represents contrary; 4: Genome collinearity of Pekin duck to Mallard, red represents the same orientation, blue represents contrary; 5: the density of SNP and Indels for Mallard, Shaoxing duck and Pekin duck in the reference Mallard genome (100 kb window). Red represents SNP, and blue represents InDels; 6: The distribution of ATAC-seq windows (100 kb window) in fat tissue; 7: The A/B compartments in Mallard genome. 8:The inner lines show syntenic blocks within the Mallard genome. **b** The genome collinearity of the genes among the three assemblies. **c** The 2,586 highly conserved genes in BUSCO dataset were used to search Mallard, Shaoxing duck and Pekin duck genomes. This analysis was carried out with the BUSCO program (version 2) with default settings. BGI_duck_1.0 and CAU_duck_1.0 are genome assemblies of Pekin duck downloaded from Genebank accessions GCA_002743455.1 and GCA_000355885.1, respectively.

All genomes were evaluated by a number of methods to validate the quality of the assemblies (Supplementary Figs. 2–6). The assembly accuracy and completeness were supported by perfect matches with 221 radiation hybrid-map marker sequences^1,11^ (Supplementary Data 1). The genomic collinearity for the three genomes shows that their assembly structure is consistent (Fig. 1a, b). The combination of a variety of sequencing technologies for these genomes significantly improved the assembly quality compared with those previously published. The contiguity of the newly assembled duck genomes (CAU_wild_1.0) is 7-fold greater than that of BGI_duck_1.0^3^ and CAU_duck_1.0 (ASM874695v1) assemblies (Supplementary Table 13). The number of gaps in the genomes also decreased to less than 0.26% (391/148961, CAU_wild_1.0 vs. BGI_duck_1.0) (Supplementary Table 13). Further improvements in genome completeness and accurate assembly of highly complex regions are also seen. The conserved genomic elements in the genome are relatively complete, based on the BUSCO scores (>95%), while the BUSCO score for the BGI_duck_1.0 assembly is less than 91% (Fig. 1c). The gene set annotated 12,061 more transcripts and 1131 more protein-coding genes than the BGI_duck_1.0 reference gene set (Supplementary Table 14). Among these newly annotated protein-coding genes, we found many functionally important genes as the high assembly quality and full-length transcriptome data were integrated into the annotation pipeline (Supplementary Table 14). Moreover, the distribution of identified TEs in the genome assemblies (average of three assemblies: 16.8%) is much more abundant than those in the previous BGI_duck_1.0 assembly (11.5%)^3^. Taken together, these results indicate that the genomes are a marked improvement in contiguity and completeness compared to the previously published duck reference genomes.

### Recovery of “missing genes” from the newly assembled genomes

The high quality of all three genome assemblies allowed us to address some classic questions in the field of evolution. Previous studies have hypothesized that the avian genome lacks some important functional genes when compared with mammals and amphibians, possibly owing to its adaptation to flight^12,13^. However, recent studies have found evidence of many of these putative “missing genes” through a more detailed genome-wide analysis^12,14,15^.

In this study, we re-checked a group of 571 genes previously thought to be missing from all avian genomes^12,13^ (Supplementary Fig. 7). We were able to identify 89 of the 571 “missing” genes, with their complete gene structure in the Mallard genome (Supplementary Table 14). It is worth noting that 5 genes were annotated as pseudogenes and 3 as lncRNA (Supplementary Table 15, Supplementary Fig. 8). In addition, 240 (42.11%) were annotated as paralogous genes (Supplementary Data 2), while 108 (18.95%) genes could be found in similar sequences in the genome but are not well annotated (Supplementary Data 3). However, 133 (23.33%) genes were still missing in the newly assembled genome with no significant sequence homologies.

**Fig. 2:**
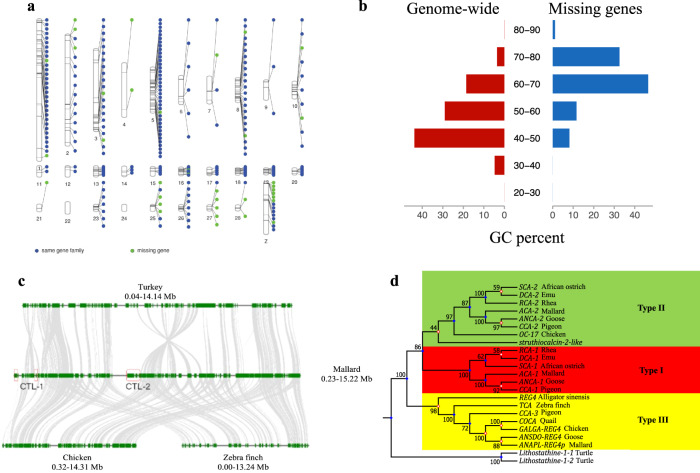
Characteristics of the CTL gene family and missing genes newly annotated in the Mallard genome. **a** The distribution of the presumed “missing genes” in the Mallard genome; **b** GC content of “missing genes” in Mallard. The figure shows that GC content distribution of missing genes in mallard was significantly higher than the genome background. **c** Multiple alignments of gene annotations in CTL gene family regions of Mallard, chicken, zebra finch, and turkey. In other bird chromosomes, the location and detailed annotations of CTL genes are lacking. **d** Phylogenetic tree of CTL members and its homologs in other birds. There are three types of CTL genes in birds, type I (green), type II (red), and type III (yellow). Ducks contain only type I and type II.

According to the distribution of the annotated “missing” genes in the Mallard genome, 66.7% of them were located on the repeat-rich/GC-rich microchromosomes (Fig. 2a, Supplementary Fig. 9). To understand more about the characteristics of these “missing genes”, we compared the GC content of 89 human-Mallard orthologs. The GC content of most (88.76%) genes is higher in Mallard than in humans, especially in the gene encoding *C2orf68*, which has a GC content of 80.59% in Mallard and only 47.29% in humans (Supplementary Fig. 10, Supplementary Table 16). Compared with other protein-coding genes in the Mallard genome, the newly discovered “missing” genes also show the characteristic of high GC content (Fig. 2b). Through quantitative analysis of gene expression, we observed that the “missing” genes have a strong tissue-specific expression in Mallard (Supplementary Fig. 11). We integrated the newly identified missing genes into the Mallard genome and previously recovered missing genes from the transcriptome assembly^15^; only 10 of all missing genes remain to be found in birds, in either the genome or transcriptome assembly (Supplementary Data 2). Therefore, our results and previous studies do not support the missing gene hypothesis in birds. We believe that the main reason that these genes have been classified as “missing” is due to either high GC content or complex genomic structure (e.g., duplications and repeat-rich regions), which has led to misassembly and/or an inability to sequence and assemble^15^.

### Identifying the avian eggshell specific CTL gene family critical for avian eggshell biomineralization

In addition to identifying “missing genes”, this genome assembly also harbors some critical genes which are unique to birds. Ovocleidin-17 (*OC-17)*, a CTL family member, is considered to be a major protein involved in eggshell calcitic mineralization in the chicken^16^. *OC-17* was the first eggshell-specific protein to be purified and to have its amino acid sequence and protein crystal structure determined^17,18^. However, in spite of the intensive effort, it has been difficult to determine the full-length cDNA and genomic DNA sequences for *OC-17* or any of its orthologs in other avian species. At present, these CTL family members are not annotated in any of the bird reference genomes. Recently, we obtained the full-length cDNA sequence of chicken *OC-17*, using transcriptome assembly and RACE^19^, and determined the expression pattern for *OC-17* in various tissues. However, its genomic sequences and the chromosomal location remains unknown in the chicken or any other avian genome.

In this study, we identified the complete gene structure of two paralogous *OC-17*-like genes (anascalcin-1, *ACA-1*; anascalcin-2, *ACA-2*); these are close together in the same chromosomal region in each of the three duck genome assemblies (Fig. 2c). In order to validate the CTL gene family annotation results in duck and to obtain CTL cDNA sequences in other avian species, we explored de novo assembled transcriptomic data from multiple tissues in five bird species (chicken, duck, pigeon, zebra finch, and goose), and performed BLAST, polymerase chain reaction (PCR), and RACE to verify the newly identified OC-17-like cDNAs. We successfully obtained 2-3 similar cDNA sequences from a pigeon (columbacalcin: *CCA-1, -2, -3*), duck (*ACA-1, -2*; ANAPL-REG4p), and goose (Ansercalcin: *ANCA-1, -2*; ANSDO-REG4), with predicted protein sequences that displayed high similarity to the *OC-17* orthologs (Supplementary Fig. 12). According to their sequence similarities, bird *OC-17*-like genes can be classified as Type-I, II, or III (Fig. 2d). The duck proteins (*ACA-1, ACA-2*) are orthologs that align with type-I and type-II sequences. There are five exons in each of the *ACA-1* and *ACA-2* genes; the length of their CDS region is 462 and 498 bp, and they encode 154 and 166 amino acids, respectively. The high GC content of the genomic sequences (*ACA-1*, 63.90%; *ACA-2*, 69.24%) and their duplication are probably responsible for the difficulty in obtaining the genomic and cDNA sequences associated with these genes. The amino acid sequence similarity between the two types of duck CTL is 56%, and their molecular weights are ~16kD. Compared with eggshell CTL protein sequences of other birds, there are 21 completely conserved amino acid sites in 16 CTL sequences, with 57 semi-conserved amino acid sites and similar secondary structures (Supplementary Figs. 12 and 13). Phylogenetic analysis indicates that the CTL family members have been duplicated multiple times during speciation (Supplementary Fig. 14). Through transcriptomic analysis, we found that expression of CTL protein-coding genes of Type-I and Type-II in Pekin duck and pigeon was highly specific to the uterine segment of the oviduct, the site of eggshell mineralization, whereas expression was negligible in the magnum (responsible for the secretion of egg white), ovary (producing the egg yolk) and other tissues (Supplementary Figs. 15 and 16). In contrast, the Type-III form in pigeons (*CCA-3*) displayed a widespread tissue-specific gene expression pattern (Supplementary Fig. 16). In the zebra finch, where our identified CTL member (Taeniocalcin, TCA) is a Type-III CTL, we observed that it is strongly expressed throughout the GI tract and at very low levels in the uterus (Supplementary Fig. 17). Expression of the Type-I and -II forms is highly specific to eggshell calcification; in the uterus where expression of the Type-I and Type-II genes is high, there is low expression of the Type-III, and vice-versa (Supplementary Fig. 15–17). We investigated the uterine expression patterns of *ACA-1* and *ACA-2* during egg formation in Pekin ducks. Tissues were collected 5–8 h before egg-laying (egg in the uterus during active eggshell calcification) and 5–9 h after egg laying (new forming egg just entering the uterus before the commencement of active calcification) (Supplementary Fig. 18). There was a stage-dependent difference in expression of each duck CTL gene, with higher expression of both *ACA-1* and *ACA-2* before egg-laying. In the uterus tissues from goose, Pekin duck, and pigeon, the expression levels before ovulation of the Type-I and Type-II CTLs are positively correlated with eggshell quality parameters such as weight (ESW), breaking strength (ESS), and thickness (EST) (Supplementary Fig. 19).

Based on genomic sequences of eggshell-specific CTL genes, we anticipate that researchers can utilize gene markers for genomic selection, conduct genome editing in cell lines or in vivo to determine the functions of CTL gene members, and gain insight into functional consequences of gene duplication during bird evolution. In summary, the duck genome assemblies from this work integrate full annotation for the eggshell CTL family genes, and we have also identified 9 cDNA sequences of CTL family members in five bird species. Moreover, this is the first instance of identifying the type-II CTL gene in the genomes of neognathae birds, since previously the simultaneous presence of Type-I and Type-II proteins have only been observed in eggshells from ratite species^20^.

### Genomic variations among Mallard and domesticated ducks

The development of high-quality duck genomes allowed us to identify large structural variants by direct comparative analysis of the three genomes. Genome comparison analysis allowed systematic characterization of presence/absence variations between the two domesticated ducks and Mallard (Supplementary Fig. 20, Supplementary Table 17, Supplementary Data 4–6). We found 350 genes in Shaoxing duck and 551 genes in Pekin duck that are located in or near these presence/absence variations regions. More than 98% of the presence/absence variations are located in the intergenic regions of these genes, with only a few of them (<2%, 12 genes for Mallard, 20 genes for Pekin duck, and 26 genes for Shaoxing duck) in exonic regions (Supplementary Data 5). Through gene enrichment analysis, we found that most of the presence/absence variations in Mallard are related to morphological development (Supplementary Data 6). In the Pekin duck, 29 of these genes are related to muscle structure development (Supplementary Data 6). By integrating our comprehensive temporal transcriptomic data from multiple-tissues/different time-points in ducks, we observed that the expression level of some genes (*DCN*, *SGCZ*, and *SGCG*) within presence/absence variations in Pekin duck are always higher than their homologs in Mallard at corresponding stages of pectoral muscle development (Supplementary Data 7). This suggests that some presence/absence variations may result in faster muscle development in the Pekin duck compared to the Mallard.

In addition, we identified genome sequence inversions between Mallard and Shaoxing duck (1.87 Mb), and between Mallard and Pekin duck (1.43 Mb) (Supplementary Data 8). We also detected 3820 translocations (1074 intra-chromosome translocations occupying 3.8 Mb, and 2746 inter-chromosome translocations occupying 6.8 Mb) (Supplementary Data 8). In total, 59% of translocations were located in the intergenic region. Our results showed that there are no large translocations and rearrangements between domestic ducks and Mallard, especially between Pekin duck and Mallard (average length 2 kb). To assess the detected SV, we verified each breakpoint by comparing Hi–C data between domestic ducks and Mallard. As a result, one significant translocation was identified at 0.72–0.81 Mb in chromosome 7 between Pekin duck and Mallard (Supplementary Fig. 21), which contains genes ENSAPLG00020017214 and ENSAPLG00020017216. The expression of gene ENSAPLG00020017214, which belongs to the *PWWP2B* protein family, in fat and muscle tissue of Pekin duck was significantly higher than in Mallard (Supplementary Data 7).

Resequencing data for 119 ducks (43 Mallards, 48 Pekin ducks, and 28 Shaoxing ducks) identified many single nucleotide polymorphisms (SNPs) within each variety: 9,232,834 (77 SNPs/kb), 12,845,466 (107 SNPs/kb), and 14,748,593 (122 SNPs/kb) in Pekin duck, Shaoxing duck, and Mallard, respectively. The total SNPs and SNP frequency in the Pekin duck genome are slightly less than that found in Shaoxing duck. We also identified small InDels: 2,711,487 (Pekin duck, 2 InDels/kb) and 4,006,473 (Shaoxing duck, 3.3 InDels/kb). Among these variants, a considerable number of SNPs were found with significantly different frequencies between domesticated ducks and Mallard. There are 311,976 SNPs that are almost fixed in Pekin duck, and 88,961 SNPs in Shaoxing duck (frequency > 0.7 in Pekin duck/Shaoxing duck, frequency < 0.3 in the Mallard; or vice versa, see “Methods”). Among these nearly fixed/lost SNPs in domestic ducks, 51,014 SNPs are located in the promoter/upstream region, while 53,598 SNPs have potential high/moderate-mutation effects as predicted by VEP (Supplementary Data 9).

### Regulatory mutations near the *NR2F2* gene affect adipose deposition

**Fig. 3:**
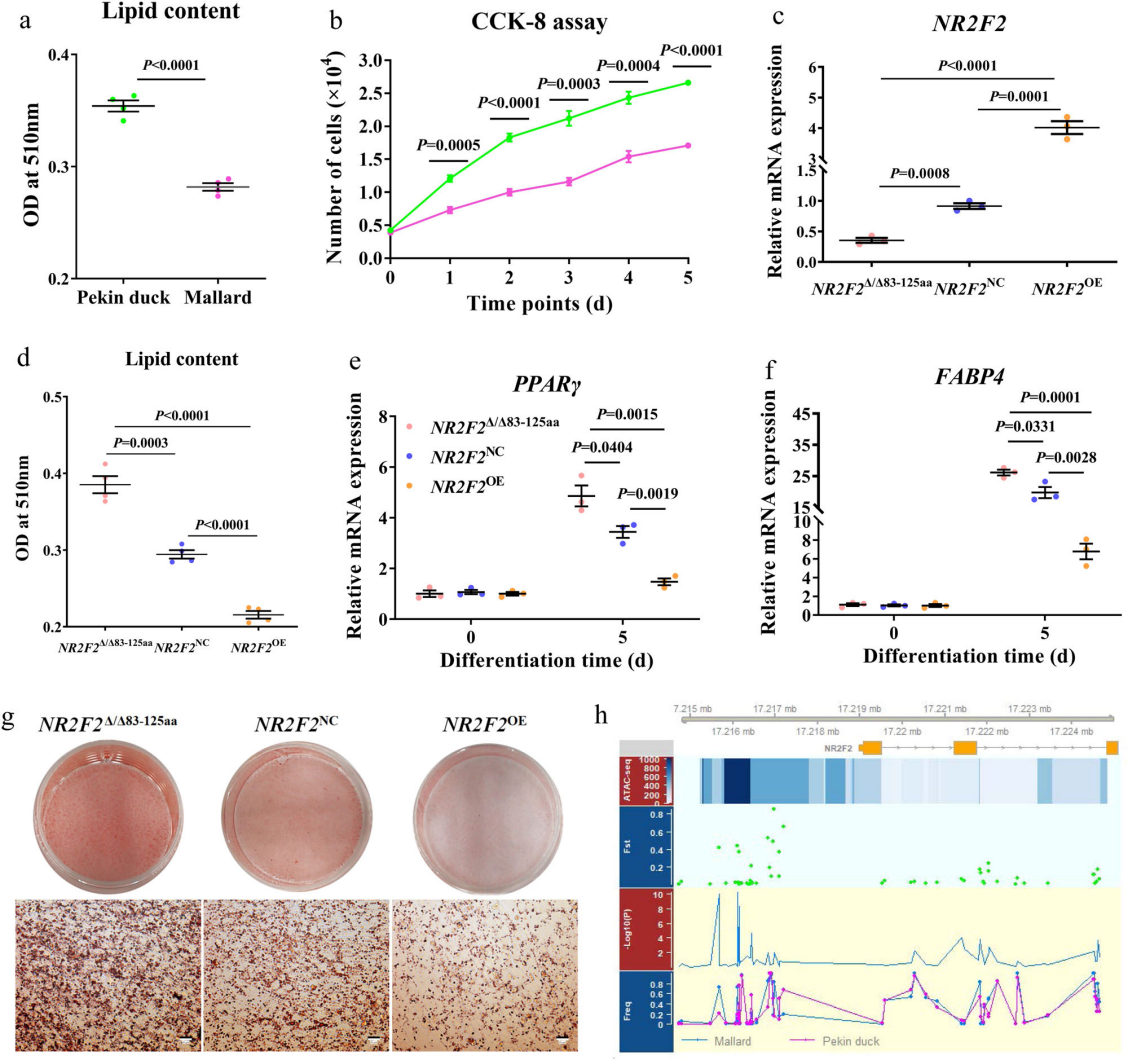
Comparison of differentiation and proliferation capacity in subcutaneous preadipocytes between Pekin duck and Mallard. **a** Intracellular lipid content in subcutaneous preadipocytes of Pekin duck and Mallard at day 5 post induction. The oil red O extraction assay was used to measure lipid accumulation. Green: Pekin duck and pink: mallard (*n* = 4 biological replicates). **b** Cell counting kit-8 assay (CCK8) examines the proliferation of subcutaneous preadipocytes in Pekin duck and Mallard over 5 days. Each cell number is counted by the standard curve established by CCK8 of the respective cells (*n* = 4 biological replicates). **c** mRNA levels of *NR2F2* were analyzed by Q-PCR in *NR2F2*^NC^, *NR2F2*^OE^, and *NR2F2*^Δ/Δ83-125aa^ cells. NC negative control, OE overexpression, Δ deleted (*n* = 3 biological replicates). **d** Intracellular lipid content in preadipocytes at day 5 post induction. The oil red O extraction assay was used to measure the lipid accumulation (*n* = 4 biological replicates). **e**, **f** mRNA levels of *PPARγ* and *FABP4* were analyzed by Q-PCR at day 0 and 5 post induction (*n* = 3 biological replicates). **g** Oil Red O staining to assess lipid accumulation at day 5 post induction for *NR2F2*^NC^, *NR2F2*^OE^, and *NR2F2*^Δ/Δ83-125aa^ cells. The scale bar represents 20 μm (*n* = 4 biological replicates). **h** The distribution of SNPs with a different frequency in *NR2F2* for Pekin duck and Mallard populations. The track below the transcript annotation represents the windows of ATAC-seq. The color depth represents the peak score size. The following tracks are shown separately: Fixation index, −log10 (*p*-value of likelihood ratio test), and allele frequency. Data are presented as mean ± SEM. Statistical significance using two-tailed unpaired Students *t*-test for (**a**–**f**).

Pekin ducks possess a subcutaneous fat weight/percentage, which is greatly elevated compared to Mallard reared in the same environment, due to intensive artificial selection during the domestication process^21,22^. Fat synthesis and deposition are coordinated processes that take place in the liver and fat tissue, respectively^23^. The growing Pekin duck has a higher liver weight (2-times heavier at 4-weeks of age) and a greater hepatic triglyceride content compared to the Mallard at 2, 4, and 6 weeks of age (Supplementary Fig. 22a, b). Similarly, the triglyceride content of subcutaneous fat tissue in Pekin duck is higher than that in Mallard, especially at 6 weeks (Supplementary Fig. 22c). The expansion of adipose deposits can be driven either by an increase in adipocyte size or by the formation of new adipocytes via precursor differentiation during adipogenesis^24^. Hence, we tested the difference in proliferation and differentiation potential for subcutaneous preadipocytes between Mallard and Pekin duck. The results showed that higher proliferation and differentiation potential are both key factors that are responsible for excessive subcutaneous fat deposition in Pekin duck (Fig. 3a, b, Supplementary Fig. 22d).

A wide array of studies have revealed the importance of transcription factors in establishing the mature fat-cell phenotype both in vitro and in vivo^24^. In order to systematically identify expression changes in transcription factors of differentiated preadipocytes cultured in vitro in the Pekin duck and Mallard, we performed mRNA-seq analysis on differentiated preadipocytes at multiple time points during adipocyte differentiation (0, 12, 24, 48, and 72 h after induction) (Supplementary Data 7). We found significant differences in the expression of most transcription factors (32/38) known to be associated with adipogenesis (Supplementary Fig. 23). Among these differentially expressed genes (DEGs), most of the transcription factors (19/24) which promote adipocyte differentiation were upregulated in Pekin duck, whereas those (14/14) that inhibit adipocyte differentiation were upregulated in Mallard. Collectively, our results indicate that differences in the expression of transcription factors play a key role in the regulation of adipocyte differentiation. We investigated this further by mRNA-Seq analysis on liver and subcutaneous fat tissue from Mallard and Pekin duck at 2, 4, and 6 weeks of age, and focused on transcription factors that are consistently differentially expressed during fat development. The transcription factors *NR3C4*, *FOXO4*, *GLIS2*, *HOXD10A*, and *RXRG*, were consistently upregulated in Pekin duck, whereas *CDX1*, *FOXC1*, *HSF1*, *NR2F2*, *TFDP1*, and *ZBTB7A* were consistently upregulated in Mallard. Notably, *NR2F2* expression in Mallard subcutaneous fat tissue, adipocytes and liver is significantly higher than in those of Pekin duck (Supplementary Fig. 24a–c), which is not seen with other transcription factors, indicating that *NR2F2* had a potential effect on subcutaneous fat deposition and metabolism during the domestication process. We investigated the fixation index (*F*_ST_) in the region around *NR2F2*, using a sliding window approach with a step of 5 kb around *NR2F2* in Pekin duck and Mallard. We found that the region located upstream of the *NR2F2* gene (Chr 12: 17.213–17.218 Mb) showed near fixation of 5 selected loci (*F*_ST_ = 0.49–0.85) from Mallard to Pekin duck (Supplementary Fig. 24d; Supplementary Data 9).

*NR2F2* was initially identified as an activator of the chicken ovalbumin gene^25^, but follow-up studies uncovered functions in adipocyte differentiation in the mouse^26^ and in metabolic gene regulation^27^; however, little is known concerning its role in birds. Therefore, we sought to investigate this further by studying the effect of mutations in *NR2F2* in immortalized chicken preadipocytes using CRISPR–Cas9. We obtained a mutant clone with a deletion of 129 bp in the first exon in both *NR2F2* alleles, located between 247 and 375 bp in the CDS region, resulting in a truncated NR2F2 protein missing 43 amino acids which included the DNA binding domain mutation, and resulted in an mRNA expression level decreased by 60% compared with the control group (Fig. 3c, Supplementary Fig. 24e). Strikingly, cells with the *NR2F2* mutation demonstrated enhanced adipogenic potential (Fig. 3d–g), as shown by increased lipid accumulation and elevated expression of adipocyte markers. On the other hand, overexpression of *NR2F2* prevented adipogenesis, further confirming an anti-adipogenic effect.

To investigate a potential regulatory role for the region defined by the five selected loci detected by Fst analysis, we performed ATAC-seq analyses to map accessible chromatin regions within this segment. We found that multiple binding sites for regulatory elements were identified in the region encompassing these five selected loci (Chr 12: 17.213–17.218 Mb) (Fig. 3h, Supplementary Table 18), indicating that this region may play an important role in the transcriptional regulation of the *NR2F2* gene and that *NR2F2* expression changes may be caused by variations in this upstream region.

However, functional verification of this potential causal mechanism is still required. Overall, our results suggest *NR2F2* as an important candidate gene that is responsible for differences in subcutaneous fat deposition between Pekin duck and Mallard.

## Discussion

In this study, we produced three high-quality genomes, for Mallard and two different breeds of domesticated ducks. Our breed-specific reference genomes provide an invaluable resource describing SNPs, indels, and structural variants, which can assist in designing specific genomic selection programs. We identified more than 36 million SNPs, indels, and structural variations that were associated with the domestication process, and these variations are now resources for illuminating the genetic differences between Mallard and domestic ducks. From a phenotype perspective, domestication has significantly improved the growth rate, reproduction ability, and fat deposition in domestic lines of ducks compared with Mallard. We do find that a large number of genes related to economic traits have undergone significant changes in domestic ducks. Among mutations in gene body regions that are predicted to have potentially high/moderate- functional effects, some mutations are known to cause specific phenotypes, i.e., an insertion in an intron region in the MITF gene causes the white feather phenotype in Pekin ducks^1^. The gene showing the most differences is *ZNF469* which has 606 SNPs between Pekin duck and Mallard, while *ZNF469* also expressed significant differences between Pekin duck and Mallard, as well as Shaoxin duck and Mallard^22^. However, we also observed that a high percentage (98.6%, 26,128/26,494) of significantly associated SNPs were located in regulatory or noncoding regions and that the mutation rate in these regulatory regions was much higher than in coding locations (Supplementary Fig. 25). Regulatory changes of this nature can be responsible for phenotypic variation, as demonstrated in a variety of organisms, including humans and chimpanzee^28,29^. However, as relevant duck cell lines are not currently available, we were not able to perform genomic-editing studies to confirm these genetic effects in vitro.

Our study demonstrates that great improvements in reference genome assembly can provide new material for the study of bird evolution. Much avian genome data have been released in recent years^30^, which allowed us to discover hundreds of “missing” genes thought to be absent from avian genomes. In our latest genome assembly, we located major missing genes on complex chromosomal regions or on micro-chromosomes, as was recently successful in the chicken assembly^31^. We have resolved the full-length genomic sequences of the eggshell CTL gene family members, which are hypothesized to be critical proteins for calcitic biomineralization in reptilia^16,17^. We also obtained the complete cDNA sequences for CTL protein members in several clades of birds. These newly discovered CTL family members expand our knowledge of eggshell biomineralization. Our preliminary tissue expression data from several species suggested potential diverse functions of different CTL isoforms in avian eggshell biomineralization, which needs to be further explored. These data should facilitate a deeper understanding of the genetic basis of calcitic biomineralization and will be a step forward in illuminating the molecular mechanisms responsible for oviparity.

## Methods

### Ethics

All experiments with birds were performed under the guidance of ethical regulations from the Animal Care and Use Committee of China Agricultural University, Beijing, China (permit number: SYXK 2007-0023).

### Sample collection, library preparation, and sequencing

#### Animals for genome assembly and population genome resequencing

We randomly selected adult female Pekin ducks, Mallard, and Shaoxing ducks from flocks that were raised under standard feeding regimes for these studies. Genome assembly: fresh blood was used for PacBio sequencing, and breast muscle tissue for Hi-C and Bionano sequencing. Forty-eight (male: 23, female: 25) 42-day-old Pekin ducks were fed with the same diet and maintained under the same lighting conditions as Golden Star Duck Co (Beijing, China), and fresh blood was collected for re-sequencing. The re-sequencing data for 43 Mallard birds were from previously published data^1^. Blood samples from the wing vein of 23 Shaoxing adult female ducks were collected in Shaoxing, China, and stored at −20 °C before DNA extraction. Information relating to sequencing samples is shown in Supplementary Data 10.

#### Library preparation and genomic sequencing for genome assembly and population genomics

Genomic DNA was extracted from the blood samples. At least 5 μg DNA was used for library construction using the Illumina TruSeq DNA Sample Prep Kit (Illumina, CA, USA). DNA was isolated using the DNeasy Blood & Tissue Kit (QIAGEN, ON, Canada). The purified genomic DNA was mechanically disrupted using Bioruptor (Diagenode Inc., NJ, USA) to generate ~300 bp inserts. The DNA fragments were subjected to end repair and A “addition to the 3” end, followed by amplification using the Thermal cycler S1000 (Bio-Rad). The purified library was subjected to quality control using StepOne Plus (Applied Biosystems, MA, USA). Finally, the Nova-seq6000 platform (Illumina, CA, USA) was used to generate paired-end sequencing data with a genome coverage of at least 10× (Supplementary Data 10).

#### Library construction and PacBio sequencing

To construct sequencing libraries for PacBio sequencing, genomic DNA was fragmented by g-TUBE centrifuged at 2000 r.p.m. for 2 min, treated with end-repair, adapter ligation and exonuclease digestion as recommended by Pacific Biosciences. DNA fragments of about 10–50 kb were selected by BluePippin electrophoresis (Sage Sciences). DNA libraries were sequenced on the PacBio Sequel platform (Pacific Biosciences).

#### Data collection for Bionano mapping

High-molecular-weight DNA was isolated and labeled according to standard BioNano protocols with the single-stranded nicking endonuclease Nt. BssSI^1,32^. The labeled DNA sample was loaded onto the IrysChip nanochannel array. The stretched DNA molecules were imaged with the BioNano Irys system. Raw image data were converted into bnx files; from these, the AutoDetect software (v2.1.4) generated basic labeling and DNA length information.

#### Hi–C library construction and data collection

We created three Hi–C libraries for Mallard, Shaoxing duck, and Pekin duck using the methods described in a similar study^1^. Libraries were subjected to sequencing on the Illumina HiSeq 2000 platform. Information relating to the raw data is given in Supplementary Data 10.

#### RNA-Seq samples, RNA-extraction, library construction, and sequencing

Uterine tissues were collected from three adult Pekin female ducks at two physiological states, before or after egg-laying, in order to analyze the expression changes in CTL genes during the daily egg formation cycle. Birds were sacrificed either 5–8 h before oviposition or 5–9 h after oviposition. Multi-tissue samples from chicken, duck, pigeon, goose, and zebra finch were collected for transcript de novo assembly, as previously described^18^. We collected liver and subcutaneous adipose tissue from Pekin duck and Mallard at 2, 4, and 6 weeks of age. Six biological replicates were collected at each time point for each group. Tissue samples were snap-frozen in liquid nitrogen and then stored at −80 °C until RNA extraction.

RNA from each tissue was extracted individually (10 μg per tissue) using Trizol reagent (Invitrogen, CA, USA) according to the manufacturer’s instructions. The Agilent Bioanalyzer 2100 instrument (Agilent, CA, USA) was used to verify the integrity of the RNA. Approximately, 10 μg of sheared cDNA was prepared for Illumina sequencing according to the manufacturer’s protocols. All samples were sequenced on the Illumina Nova-seq6000 system (Illumina, CA, USA) with 150 bp paired ends. Pekin duck full-length transcriptome data were obtained from our previously published dataset^33^ (PRJNA526109).

### Genome assembly

Falcon^34^ was used for constructing initial contigs using the following parameters: length_cutoff = 13,000 length_cutoff_pr = 14,000 pa_DBsplit_option = -x1000 -s250 -a pa_HPCdaligner_option = -v -dal128 -t12 -e.75 -k20 -h320 -l1800 -s1000 falcon_sense_option = --output_multi --min_idt 0.75 --min_cov 2 --local_match_count_threshold 2 --max_n_read 400 --output_dformat ovlp_DBsplit_option = -x1000 -s200 ovlp_HPCdaligner_option = -v -dal100 -t12 -k18 -h280 -e.96 -l1800 -s1000 overlap_filtering_setting = --max_diff 50 --max_cov 80 --min_cov 2 --bestn 10. The initial polishing was performed with Quiver^35^ using PacBio-only long reads, and then Pilon^36^ (v1.20) was utilized to further correct the PacBio-corrected contigs with accurate Illumina short reads. The BioNano data was first assembled into a consensus map using the IrysView software (with default parameters) with a molecular length threshold of 150 kb and a minimum labels per molecule of 8. Hybrid scaffolding of the PacBio-corrected contigs and the BioNano-based consensus map was performed using the hybrid scaffolding module within IrysView software with the manufacturer’s suggested parameters. After scaffolding, PBJelly from PBSuite^37^ (v14.9.9) was performed to close gaps in the hybrid assembly. We re-performed error correction procedures to polish the sequences in the gap regions. Subsequently, the mitochondrial scaffolds or contigs were removed through alignment to mitochondrial references (BGI_duck_1.0); any scaffolds or contigs for which at least 80% of the total length was aligned and that showed an identity larger than 90% were discarded as mitochondrial sequences. The Hi–C sequencing data were first aligned to the assembled contigs/scaffolds using the Bowtie2^38^ end-to-end algorithm, and then the assembled scaffolds were clustered, ordered, and directed into pseudochromosomes using Lachesis^39^. Finally, the pseudo-chromosomes predicted by Lachesis^39^ were cut into bins with an equal length of 100 kb and used to construct a heatmap based on the interaction signals generated by valid mapped read pairs to perform validation and manual correction. The final draft was corrected using Gapcloser^40^ with default parameters. The 2586 conserved protein models in the BUSCO vertebrata_odb9 dataset were searched against all assembled genomes by using the BUSCO (version 2) program with default settings^41^.

### Genome annotation

Annotation of the Mallard assembly (ASM874695v1) was created via the Ensembl gene annotation system. A set of potential transcripts was generated using two major techniques: primarily through alignment of short-read RNA-seq data and also through gap filling with protein-to-genome alignments of a subset of vertebrate proteins with experimental evidence from UniProt^42^ vertebrate proteins. The short-read RNA-seq data was sourced from various samples generated as part of these projects (PRJNA194464 and PRJNA273367)^3,15^. The UniProt vertebrate proteins had experimental evidence for existence at the protein or transcript level. At each locus, low-quality transcript models were removed, and the data were collapsed and consolidated into a final gene model plus its associated non-redundant transcript set. When collapsing the data, priority was given to models derived from transcriptomic data. For each putative transcript, the coverage of the longest open reading frame was assessed in relation to known vertebrate proteins, to help differentiate between true isoforms and fragments. In loci where the RNA-seq data were fragmented or missing, homology data took precedence, with preference given to longer transcripts that had strong intron support from the short-read data. Gene models from the above process were classified into three main types: protein-coding, pseudogene, and long noncoding RNA. Models with hits to known proteins and few structural abnormalities (i.e., canonical splice sites, introns exceeding a minimum size threshold, low level of repeat coverage) were classified as protein-coding. Models with hits to known protein, but having multiple issues in their underlying structure were classified as pseudogenes. Single-exon models, with a corresponding multi-exon copy elsewhere in the genome, were classified as processed pseudogenes. If a model failed to meet the criteria of any of the previously described categories, did not overlap a protein-coding gene, and had been constructed from transcriptomic data, then it was considered as a potential lncRNA. Potential lncRNAs were filtered to remove transcripts that did not have at least two valid splice sites or cover 1000 bp (to remove transcriptional noise). Using LastZ^43^, we generated a whole-genome alignment against the human assembly GRCh38.p12. For each protein-coding gene in the human (Ensembl/GENCODE gene set), we projected the coding exons within the canonical transcript to the Mallard. In the case of an exonic overlap on the projected sequence, the longest exon took precedence. If the mapping did not succeed, we selected the next successful projection of the transcript having the longest translation. The annotation of small noncoding genes, particularly miRNAs, were annotated via a BLAST^44^ of miRbase^45^ against the genome, before passing the results into RNAfold^46^. Poor quality and repeat-ridden alignments were discarded. Other types of small noncoding genes were annotated by scanning Rfam^47^ against the genome and passing the results into Infernal^48^.

The genome assemblies of Pekin duck, (JACEUL000000000) and Shaoxing duck (JACEUM000000000) were annotated using the EVidenceModeler (EVM) pipeline^49^. In the homology annotation, Genewise (2.4.1)^49^ was used to map the gene sets of Turkey, Chicken, Ostrich, and Zebra finch to the assembled genomes for homology prediction. Augustus (2.5.5)^50^, GlimmerHMM (3.0.4)^51^, SNAP^52^, Geneid(v 1.4.4)^53^, and Genscan^54^ were used to de novo predict gene structure. In addition, we used full-length transcriptome data to predict the structure of the transcripts by Pasa (2.3.1)^55^ and Cufflink (2.2.1)^56^. Finally, the EVidenceModeler (v1.1.1)^49^ was used to integrate the prediction from all sources. The gene sets predicted by the EVM process were searched against public protein databases, using the Blast algorithm, for annotation, including UniProt^42^, Nr^57^, pfam^58^, GO^59,60^, KEGG^61^, and InterPro^62^ (Supplementary Table 8).

### Annotation of repeats

We searched for repetitive sequences in the duck genomes, including tandem repeats and transposable elements (TEs). Tandem Repeats Finder^63^ (TRF, v4.09) was employed to annotate the tandem repeats with the following parameters: 2 7 7 80 10 50 2000. Then the TEs were identified at both the DNA and protein levels using a combination of de novo and homology-based approaches. At the DNA level, LTR_FINDER^64^(v1.0.6) was first used to identify LTR-RTs, and RepeatModeler^65^ (v1.0.5) was utilized to construct a de novo repeat library, which comprised a repeat consensus database with classification information. We employed RepeatMasker^65^ (v4.0.6) to search for similar TEs in the known Repbase TE library^66^ and the de novo repeat library. At the protein level, RepeatProteinMask within the RepeatMasker package was used to search against the TE protein database using a WU-BLASTX engine.

### Genome alignment and gene synteny analysis

Genome alignment among the three duck genomes was performed using the MUMmer^67^ (version 4.00beta2) program with parameter settings –maxmatch –c 90 –l 40. The alignments were filtered by running delta-filter with parameter −1. SNPs and InDels in the two accessions were extracted by running show-diff in the one-to-one alignment blocks. We also mapped DNA sequencing data (>10×) from the Illumina HiSeq platform for each accession against the other genomes using BWA (version 0.7.10- r789) software. All these variants were annotated using the VEP program^68^. To identify syntenic gene blocks among the duck genomes, we conducted an All-vs.-All blastp (*e*-value < 1e−10, -v 5, -b 5) for each genome pair. The homologous genes were analyzed by the MCScanX package^69^ with default settings, except for gap_penalty -3. Syntenic blocks were defined as those with at least five syntenic genes.

### Identification of inversions and translocations

The genomes of Shaoxing and Pekin ducks were aligned with Mallard using MUMmer4^67^ (version 4.00beta2) to identify inversions and translocations. The alignment blocks exhibiting inversions were extracted for manual checking. Alignment blocks identified in different positions were extracted to check their flanking blocks. If alignment blocks had non-colinear flanking sequences, those were retained as putative translocations. The translocations were further divided into inter-chromosomal translocations and intra-chromosomal translocations. Both inversions and translocations were identified if they possessed a length > 100 bp and an identity > 90%.

### Identification of presence/absence variations

Putative presence/absence variations were identified by extracting unaligned regions between Mallard and domestic ducks from the “show-diff” command in MUMmer4^67^ (version 4.00beta2). This approach gave sequences of 8.14 Mb for Mallard, and 7.55 Mb for Shaoxing duck between Mallard and Shaoxing duck, in addition to 4.41 Mb for Mallard and 12.03 Mb for Pekin duck between Mallard and Pekin duck. These sequences were then filtered by discarding those overlapping with gap regions in the respective genome. The remaining sequences above were then filtered by alignment with the contig and scaffold of corresponding genomes using blastn (1e-5) to identify putatively unique regions. The segments with coverage > 50%, and identity > 90% were filtered. Finally, the whole genome re-sequencing data were mapped to the respective genome to confirm the potential presence/absence variations. The one-tailed *t* test was used to determine whether the coverage was significantly different between domestic ducks and Mallard.

### SNP/indel calling

The variant calling was performed using the Speedseq pipeline^70^. After trimming of low-quality bases using Trimmomatic (version 0.32), the clean data were mapped to the Mallard genome using BWA software^71^. The re-sequencing data for Mallard were downloaded from the NCBI Sequence Read Archive (SRA) database. The clean data were mapped to the wild CAU_wild_1.0 genome using BWA software^71^. All the unique mapping data were extracted to identify SNPs and small InDels using freebayes and Samtools^72^ (version 0.1.19) programs. Variants were removed with QualByDepth (QD) < 4.0, 300 > depth > 2200, Quality < 30, mapping quality (MQ) < 40.0, MQRankSum < -10, Read- PosRankSum < -7.0, Fisher Strand > 60.0, ReadPosRankSum > 7, BaseQRankSum < -6, BaseQRankSum > 6”. Cluster Size and ClusterWindowSize were set to 4 and 10, respectively. For the subsequent analyses, we used only bi-allelic SNPs on autosomes. VEP^73^ was used to annotate variants according to their functional categorization, which included the following categories: 5 kb up- and downstream of a gene, intergenic, missense, synonymous, intronic, 3′ untranslated regions, 5′ untranslated regions, stop gain and stop loss. Variants in the up- and downstream regions and in the 3′ UTR/5′ UTR regions were merged into single categories.

### Strategy to identify missing genes

We used the annotated transcripts of the Mallard genome to find sequences homologous to any of the 571 genes previously thought to be missing from the bird genome^13,30^, of which 274 were thought to be missing from all avian genomes. The human protein sequences of the corresponding missing genes were used as query sequences to search for homologies in the newly assembled Mallard genome using the best-reciprocal blast algorithm. We manually checked each matched candidate sequence based on the list of missing genes to distinguish matching paralogous products and alignment errors.

### Detection of selective sweeps

To detect putative selective sweeps, we first searched the genome for regions with high degrees of differentiation between Mallard and domestic lines. We estimated fixation index (*F*_ST_)^74^, and *p*-value of likelihood ratio test using vcflibs (https://github.com/vcflib/vcflib#vcflib).

### RNA-seq analysis

Raw reads were trimmed to remove adapters and low-quality reads, with Trimmomatic (version 0.39)^75^. Trimmed reads were mapped to the Mallard genome using HISAT2^76^. Read counts for each gene were calculated using HTSeq^77^ and normalized by library sequencing depth using the TMM method implemented in DESeq2 (v.1.24.0)^78^, after filtering genes with no expression. We used DESeq2 to identify DEGs between Mallard and Pekin duck tissues at different times. Samples that had an average *R*^2^ (the square of the Pearson correlation coefficient) greater than 0.95 when compared with other samples, were accepted as valid biological replicates. Genes with |log2FC | > 0.584 and the Benjamini–Hochberg (BH) adjusted *p*-value (adjusted-*p* value) < 0.05 were considered as DEGs.

The de novo assembled transcripts of active uterine tissues during the laying period for five bird species (chicken, duck, goose, pigeon, and zebra finch) were obtained from our previous study^15^. We used BLASTX (*E* = 1e−10) to seek the orthologs of chicken and emu OC-17-like transcripts in the assembled transcript dataset. Once we obtained the best candidate transcripts, we designed primers for each candidate transcript of each species to obtain the cDNA.

### Functional annotation and enrichment

Gene enrichment analysis for structural variations (within the gene or ~5 kb in the up/downstream of gene) and DEGs were completed with the Metascape^79^ (2019-8-14) software using the human reference genome to assign genes to the corresponding terms. Enrichment tests were performed using the hypergeometric test and Benjamini–Hochberg *p*-value correction algorithm as described in Metascape.

### RACE (rapid amplification of cDNA ends) for cloning CTL gene family members

5′ and 3′ RACE were performed using the Smarter^®^ RACE 5′/3′ Kit (Takara Bio Inc., USA) according to the manufacturer′s instructions. RACE-PCR products were obtained with SeqAmp DNA Polymerase (Takara Bio Inc., USA) using the Universal Primer Mix (supplied) and a gene-specific primer (Supplementary Table 19). Products were visualized on a 2% agarose gel and purified by NucleoSpin Gel and PCR Clean-UpKit (Takara Bio Inc, USA). This product was then subcloned into the In-Fusion HD Cloning vector (Takara Bio Inc., USA) and grown in TOP10 *E. coli*. Clones were sequenced with the M13 forward primer.

### Phylogenetic analysis for CTL gene families

The known bird eggshell CTL proteins were downloaded from Uniprot (http://www.uniprot.org/), including chicken Ovocleidin-17 (*OC-17*, Q9PRS8), African ostrich Struthiocalcin-1 (*SCA-1*, P83514) and Struthiocalcin-2 (*SCA-2*, P83515), rhea Rheacalcin-1 (*RCA-1*, P84617), and Rheacalcin-2 (*RCA-2*, P84618), emu Dromaiocalcin-1 (*DCA-1*, P84615), Dromaiocalcin-2 (*DCA-2*, P84616), Green sea-turtle Lithostathine-1-1 (UY3_13503, M7B1U1), and Green sea-turtle Lithostathine-1-2 (UY3_02957, M7BRJ1). The nine cDNA sequences generated in this study were conceptually translated into amino acid sequences and used for the sequence alignments. The CTL proteins translated from the cDNA of this study were blasted against the UniProtKB database. A total of 119 amino acid sequences of representative species in different classes (fishes, amphibians, reptiles, birds, and mammals) were selected from the blast results (*E*-value < 1e−10, Identity > 25%). All sequence information is listed in Supplementary Data 11.

Multiple alignments of the amino acid sequences were done by the MUSCLE algorithm implemented in MEGA^80^ (version 10) (MUSCLE, Max Iteration = 8). A phylogenetic tree was constructed using the JTT + G + I model with 100 bootstraps, with the purple sea urchin (echinoderma) used as the outgroup. The crystal structure files of African ostrich Struthiocalcin-1(4UWW.pdb) and chicken Ovocleidin-17(1GZ2.pdb) were obtained from the RCSB PDB (https://www.rcsb.org/) website. ESPript (version 3.0)^81^ was used to show the conserved and structural characteristics of the CTL gene family (parameters: MODE, ADV; Strict Global score: 0.5).

### ATAC-seq sequencing and analysis of NR2F2 promoter binding region

Fifty thousand nuclei from Pekin duck subcutaneous preadipocytes (*N* = 2) before and after oleic acid-induced adipogenic differentiation were transposed using Tn5 transposase as previously described^82^. Briefly, cells were lysed using ice-cold lysis buffer (10 mM Tris-HCl pH = 7.4, 10 mM NaCl, 3 mM MgCl_2_ and 0.1% IGEPAL CA-630) and centrifuged at 2400 r.p.m. (500×*g*) for 10 min. The pellet was resuspended in the transposase reaction mix and incubated at 37°C for 30 min. The sample was column-purified and amplified by 15 cycles of PCR before high-throughput sequencing. Each dataset was aligned to the Mallard genome using Bowtie2^38^. After alignment, each group of replicates were merged together, sorted, and indexed. Duplicated reads and low mapping quality reads (mapping score <30) were removed. The merged, filtered, and sorted BAM files were used as the input for HMMRATAC (default parameters)^83^. The motif analysis and annotation were performed using the Homer toolkit^82^.

### Adipogenic transcriptional regulatory network

The mapping of the adipogenic gene regulatory network is based on the established white fat differentiation cascade regulatory pathway as described in WikiPathways and recent reviews^84–86^ (Supplementary Fig. 23a). We then superimposed the expression information as determined from RNA-seq (fold changes compared to day 0 and multiple-testing corrections) on the network (Supplementary Fig. 23a, b).

### Isolation of stromal vascular cells (preadipocytes) from duck subcutaneous fat tissue biopsies

Preadipocytes were obtained from Pekin duck and Mallard using the same protocol^87^. First, isolated subcutaneous adipose tissues were cut into small pieces of about 1 mm^3^ and digested with 1 mg/mL collagenase A (Sigma-Aldrich, MO, USA) in DMEM/F12 (Dulbecco’s modified Eagle’s medium/Ham’s nutrient mixture F-12, Gibco, Gaithersburg, MD, USA) supplemented with 4% BSA, 100 mM Hepes and 150 nM adenosine (Sigma-Aldrich, MO, USA) for 70 min at 37 °C in a shaking water bath. The digest was filtered through nylon screens with 70 μm mesh openings, and the mixture was centrifuged at 1500 × *g* for 10 min to remove mature adipocytes and obtain adipose-derived stromal cells. Finally, cells were resuspended in DMEM/F12 supplemented with 10% FBS and 1% antibiotic/antimycotic solution (Gibco, Gaithersburg, MD, USA) for further manipulation.

### Plasmids for constructing NR2F2-editing cell lines

Using the *chNR2F2* sequence obtained from the NCBI database (Accession: NC_007129.7), we designed gRNA sequences targeting exon1 of *chNR2F2*, known as sgRNA1: GTTTGTGGGGACAAGTCTAG and sgRNA2: GGCAGTACTGACACTGATTG. We synthesized the oligo-DNAs corresponding to these gRNA sequences and annealed them to a T7 promoter-driven Cas9 vector and to a U6 promoter-driven gRNA vector in order to obtain two gRNA-expressing plasmids. In order to construct the *NR2F2*-overexpression vector, the full-length coding sequence of *chNR2F2* (Gene ID: 386585) was amplified from chicken subcutaneous adipose cDNA by PCR, and cloned into the CMV promoter-driven piggyBac and an EF1α promoter-driven GFP plasmid by replacing GFP using *Eco*RI and *Sal*I (New England Biolabs, Ipswich, MA, USA). All plasmids in this study were a gift from Professor S. Wu (State Key Laboratory of Agrobiotechnology, College of Biological Sciences, China Agricultural University). All cloning plasmids were confirmed by sequencing.

### Cell culture and transfection of the immortalized chicken preadipocyte cell line

A cell line of immortalized chicken preadipocytes (ICP1)^88^ was a kind gift of the Poultry Breeding Group of the College of Animal Science and Technology, Northeast Agricultural University, China, and was cultured in DMEM/F12 cell culture medium with 10% FBS, at 37 °C with 5% CO_2_. ICP1 preadipocytes were seeded in 6-well plates for further transfection using Lipofectamine 2000 (Invitrogen), and the transfection procedure was performed according to the manufacturer’s instructions. After a 48 h recovery period, the cells were supplemented with 3 μg/mL of puromycin (Sigma-Aldrich, MO, USA) to screen out cells which have not been successfully transfected into the plasmids (ie, negative cells). Once the cell clone is formed, cells were harvested using 0.25% trypsin/EDTA (Gibco, Gaithersburg, MD, USA), and the cell density was calculated using a handheld automated cell counter (Millipore, Darmstadt, Germany). Single cells were plated in each well of a 96-well plate by limiting dilution and then cultured for 10 d in the cell culture medium. The medium was replaced every 4 d. Confluent cell colonies were propagated and genotyped by PCR and sequencing. Primer sets used for PCR are listed in Supplementary Table 19.

### Adipogenic differentiation of duck preadipocytes and ICP1 cells

Adipogenic differentiation of duck subcutaneous preadipocytes and ICP1 cells were induced using the same protocol^89^. The duck preadipocytes and ICP1 cells were expanded in culture using DMEM/F12 cell culture medium with 10% FBS. Cells at passage three to four were induced to differentiate after 2 days of confluence (day 0) with 300 nM oleic acid (Sigma-Aldrich, MO, USA) in DMEM/F12 supplemented with 10% FBS, and 1% antibiotic solution. After 3 days, the medium was changed to a cell culture medium (DMEM/F12 with 10% FBS). The medium was changed every 2 days throughout the differentiation period. Cells were fixed with 10% formalin for 20 min and stained with Oil Red O (Sigma-Aldrich, MO, USA) to examine lipid accumulation. After another wash with PBS, the cell nuclei were counterstained with Hoechst 33342 (Sigma-Aldrich, MO, USA). All experiments were repeated three times, and samples were treated in triplicate. Morphological changes were observed and photographed under an inverted fluorescent microscope (Nikon). Lipid droplet accumulation was measured by the Oil Red O extraction assay, as described by Ramirez et al.^90^.

### Q-PCR analysis

Total RNA was isolated from cells with the EZNA total RNA kit (Omega Bio-Tek, GA, USA) according to the manufacturer’s instructions. Quantification of RNA was performed with the Nanodrop 2000 Spectrophotometer (Thermo Fisher Scientific, MA, USA). RNA was reverse transcribed using the PrimeScript RT Master Mix kit (Takara Bio, USA), and used in quantitative PCR reactions containing SYBR-green fluorescent dye (Applied Biosystems, MA, USA). Q-PCR was performed using the ABI-7500 PCR machine. Gene-specific primers were designed using Primer 3 software (version 0.4.0, Howard Hughes Medical Institute). Primer sets are listed in Supplementary Table 40. The relative expression of mRNAs was determined after normalization with GAPDH levels using the 2^−^^ΔΔ^Ct method^91^.

### Cell count kit 8 assay for duck preadipocyte proliferation

Pekin duck and Mallard subcutaneous preadipocytes (4000 per well) were cultivated in 96-well plates, and cell proliferation was detected after 6 days with the cell counting kit-8 (Dojindo, Kumamoto, Japan) at 450 nm using a Model 680 Microplate Reader (Bio-Rad). All the data were acquired by averaging the results from four independent experiments.

### Triglyceride concentration assay for duck liver and fat tissue

The liver and fat tissue homogenates were digested in RIPA buffer (Thermo Fisher Scientific, MA, USA) containing protease inhibitor cocktail (Sigma-Aldrich, MO, USA), before measurement of protein content using BCA Protein Assay Kit (Sigma-Aldrich, MO, USA). Total triglyceride levels were measured using a Triglyceride Reagent kit (Sigma-Aldrich, MO, USA).

### Reporting summary

Further information on research design is available in the Nature Research Reporting Summary linked to this article.

## Supplementary information

Supplementary information

Description of Additional Supplementary Files

Supplementary Data 1

Supplementary Data 2

Supplementary Data 3

Supplementary Data 4

Supplementary Data 5

Supplementary Data 6

Supplementary Data 7

Supplementary Data 8

Supplementary Data 9

Supplementary Data 10

Supplementary Data 11

Reporting Summary

## Data Availability

genome assembly datasets reported in this study have been deposited in GenBank (NCBI,) and BIG with the following accession codes: Mallard genome, PRJNA554956; Pekin duck genome, (GenBank, JACEUL000000000; BIG, GWHANUR00000000); Shaoxing duck genome (GenBank, JACEUM000000000; BIG, GWHANUS00000000). All datasets have also been deposited in the Genome Warehouse of the BIG Data Center at the Beijing Institute of Genomics, Chinese Academy of Sciences (https://ngdc.cncb.ac.cn/bioproject/), under the following accession numbers: whole-genome re-sequencing data, CRA002746; whole-genome sequencing data of Shaoxing duck, CRA002750 and CRA002733; dynamic transcriptome sequencing of Mallard liver tissue, CRA002743; dynamic transcriptome sequencing of Mallard skin fat tissue, CRA002755; dynamic transcriptome sequencing of Pekin duck liver tissue, CRA002747; dynamic transcriptome sequencing of Pekin duck skin fat tissue, CRA002754; RNA-Seq of Mallard subcutaneous preadipocytes, CRA002775. The RNA-Seq of Pekin duck subcutaneous preadipocytes^92^, the Iso-Seq of Pekin duck^33^, and whole-genome re-sequencing of Mallard^1^ have been reported previously, and the data were deposited into the NCBI database under accession numbers SRX4646736, SRP188279, PRJNA450892, respectively. The raw sequencing data also reported in this paper have been deposited in the Sequence Read Archive (SRA, https://www.ncbi.nlm.nih.gov/sra) under NCBI BioProject accession PRJNA645648 and PRJNA554956. The sequence source of the public database were shown below: Uniprot database was downloaded from https://www.uniprot.org/; Ensembl/GENCODE gene set of human was downloaded from http://ftp.ensembl.org/pub/release-103/fasta/homo_sapiens/pep/Homo_sapiens.GRCh38.pep.all.fa.gz; Nr database was downloaded from https://ftp.ncbi.nlm.nih.gov/blast/db/FASTA/nr.gz; KEGG database was downloaded from https://www.genome.jp/kegg/; InterPro was downloaded from https://www.ebi.ac.uk/interpro/; Pfam database was downloaded from http://pfam.xfam.org/; GO database was downloaded from http://geneontology.org/. All data and research materials are available upon reasonable request by contacting the corresponding author.

## References

[1] Zhou Z (2018). An intercross population study reveals genes associated with body size and plumage color in ducks. Nat. Commun..

[2] Zhang ZB (2018). Whole-genome resequencing reveals signatures of selection and timing of duck domestication. Gigascience.

[3] Huang Y (2013). The duck genome and transcriptome provide insight into an avian influenza virus reservoir species. Nat. Genet..

[4] Olsen B (2006). Global patterns of influenza a virus in wild birds. Science.

[5] Venkatesh D (2018). Avian influenza viruses in wild birds: virus evolution in a multihost ecosystem. J. Virol..

[6] Lawal RA (2020). The wild species genome ancestry of domestic chickens. BMC Biol..

[7] Piegu B (2020). Variations in genome size between wild and domesticated lineages of fowls belonging to the *Gallus gallus* species. Genomics.

[8] Tian X (2020). Building a sequence map of the pig pan-genome from multiple de novo assemblies and Hi-C data. Sci China Life Sci..

[9] Hincke MT (2012). The eggshell: structure, composition and mineralization. Front. Biosci..

[10] Erben HK, Hoefs J, Wedepohl KH (1979). Paleobiological and isotopic studies of eggshells from a declining dinosaur species. Paleobiology.

[11] Rao M (2012). A duck RH panel and its potential for assisting NGS genome assembly. BMC Genomics.

[12] Zhang G (2014). Comparative genomics reveals insights into avian genome evolution and adaptation. Science.

[13] Lovell PV (2014). Conserved syntenic clusters of protein coding genes are missing in birds. Genome Biol..

[14] Botero-Castro F, Figuet E, Tilak MK, Nabholz B, Galtier N (2017). Avian genomes revisited: hidden genes uncovered and the rates versus traits paradox in birds. Mol. Biol. Evol..

[15] Yin ZT (2019). Revisiting avian ‘missing’ genes from de novo assembled transcripts. BMC Genomics.

[16] Reyes-Grajeda JP, Moreno A, Romero A (2004). Crystal structure of ovocleidin-17, a major protein of the calcified *Gallus gallus* eggshell: implications in the calcite mineral growth pattern. J. Biol. Chem..

[17] Hincke MT, Tsang CP, Courtney M, Hill V, Narbaitz R (1995). Purification and immunochemistry of a soluble matrix protein of the chicken eggshell (ovocleidin 17). Calcif. Tissue Int..

[18] Mann K, Siedler F (1999). The amino acid sequence of ovocleidin 17, a major protein of the avian eggshell calcified layer. Biochem. Mol. Biol. Int..

[19] Zhang Q (2014). Integrating de novo transcriptome assembly and cloning to obtain chicken Ovocleidin-17 full-length cDNA. PLoS ONE.

[20] Mann K, Siedler F (2004). Ostrich (*Struthio camelus*) eggshell matrix contains two different C-type lectin-like proteins. Isolation, amino acid sequence, and posttranslational modifications. Biochim. Biophys. Acta.

[21] Fan W (2020). Dynamic accumulation of fatty acids in duck (Anas platyrhynchos) breast muscle and its correlations with gene expression. BMC Genomics.

[22] Chen L (2015). Transcriptome analysis of adiposity in domestic ducks by transcriptomic comparison with their wild counterparts. Anim. Genet..

[23] Goodridge AG, Ball EG (1967). Lipogenesis in the pigeon: in vivo studies. Am. J. Physiol..

[24] Ghaben AL, Scherer PE (2019). Adipogenesis and metabolic health. Nat. Rev. Mol. Cell Biol..

[25] Knoll BJ, Zarucki-Schulz T, Dean DC, O’Malley BW (1983). Definition of the ovalbumin gene promoter by transfer of an ovalglobin fusion gene into cultured cells. Nucleic Acids Res..

[26] Xu Z, Yu S, Hsu CH, Eguchi J, Rosen ED (2008). The orphan nuclear receptor chicken ovalbumin upstream promoter-transcription factor II is a critical regulator of adipogenesis. Proc. Natl Acad. Sci. USA.

[27] Ashraf UM, Sanchez ER, Kumarasamy S (2019). COUP-TFII revisited: its role in metabolic gene regulation. Steroids.

[28] Franchini LF, Pollard KS (2017). Human evolution: the non-coding revolution. BMC Biol..

[29] Hubisz MJ, Pollard KS (2014). Exploring the genesis and functions of Human Accelerated Regions sheds light on their role in human evolution. Curr. Opin. Genet. Dev..

[30] Zhang G (2015). Genomics: bird sequencing project takes off. Nature.

[31] Warren WC (2017). A new chicken genome assembly provides insight into avian genome structure. G3 (Bethesda).

[32] Lam ET (2012). Genome mapping on nanochannel arrays for structural variation analysis and sequence assembly. Nat. Biotechnol..

[33] Yin ZT, Zhang F, Smith J, Kou R, Hou ZC (2019). Full-length transcriptome sequencing from multiple tissues of duck, *Anas platyrhynchos*. Sci. Data.

[34] Chin CS (2016). Phased diploid genome assembly with single-molecule real-time sequencing. Nat. Methods.

[35] Chin CS (2013). Nonhybrid, finished microbial genome assemblies from long-read SMRT sequencing data. Nat. Methods.

[36] Walker BJ (2014). Pilon: an integrated tool for comprehensive microbial variant detection and genome assembly improvement. PLoS ONE.

[37] English AC (2012). Mind the gap: upgrading genomes with Pacific Biosciences RS long-read sequencing technology. PLoS ONE.

[38] Langmead B, Salzberg SL (2012). Fast gapped-read alignment with Bowtie 2. Nat. Methods.

[39] Burton JN (2013). Chromosome-scale scaffolding of de novo genome assemblies based on chromatin interactions. Nat. Biotechnol..

[40] Xu GC (2019). LR_Gapcloser: a tiling path-based gap closer that uses long reads to complete genome assembly. Gigascience.

[41] Waterhouse RM, Seppey M, Simao FA, Zdobnov EM (2019). Using BUSCO to assess insect genomic resources. Methods Mol. Biol..

[42] McGinnis W, Levine MS, Hafen E, Kuroiwa A, Gehring WJ (1984). A conserved DNA sequence in homoeotic genes of the Drosophila Antennapedia and bithorax complexes. Nature.

[43] Harris, R.S. Improved pairwise alignment of genomic DNA. *Ph.D. thesis*, The Pennsylvania State University (2007).

[44] Altschul SF, Gish W, Miller W, Myers EW, Lipman DJ (1990). Basic local alignment search tool. J. Mol. Biol..

[45] Kozomara A, Birgaoanu M, Griffiths-Jones S (2019). miRBase: from microRNA sequences to function. Nucleic Acids Res..

[46] Gruber AR, Lorenz R, Bernhart SH, Neubock R, Hofacker IL (2008). The Vienna RNA websuite. Nucleic Acids Res..

[47] Kalvari I (2018). Rfam 13.0: shifting to a genome-centric resource for non-coding RNA families. Nucleic Acids Res..

[48] Nawrocki EP, Eddy SR (2013). Infernal 1.1: 100-fold faster RNA homology searches. Bioinformatics.

[49] Haas BJ (2008). Automated eukaryotic gene structure annotation using EVidenceModeler and the Program to Assemble Spliced Alignments. Genome Biol..

[50] Stanke M (2006). AUGUSTUS: ab initio prediction of alternative transcripts. Nucleic Acids Res..

[51] Majoros WH, Pertea M, Salzberg SL (2004). TigrScan and GlimmerHMM: two open source ab initio eukaryotic gene-finders. Bioinformatics.

[52] Bromberg Y, Rost B (2007). SNAP: predict effect of non-synonymous polymorphisms on function. Nucleic Acids Res..

[53] Blanco, E., Parra, G. & Guigo, R. Using geneid to identify genes. *Curr. Protoc. Bioinform*. Chapter 4, Unit 4 **3** (2007). 10.1002/0471250953.bi0403s18 18428791

[54] Burge C, Karlin S (1997). Prediction of complete gene structures in human genomic DNA. J. Mol. Biol..

[55] Haas BJ (2003). Improving the Arabidopsis genome annotation using maximal transcript alignment assemblies. Nucleic Acids Res..

[56] Ghosh S, Chan CK (2016). Analysis of RNA-Seq data using TopHat and cufflinks. Methods Mol. Biol..

[57] Coordinators NR (2018). Database resources of the National Center for Biotechnology Information. Nucleic Acids Res..

[58] El-Gebali S (2019). The Pfam protein families database in 2019. Nucleic Acids Res..

[59] The Gene Ontology, C. (2019). The Gene Ontology Resource: 20 years and still GOing strong. Nucleic Acids Res..

[60] Ashburner M (2000). Gene ontology: tool for the unification of biology. The Gene Ontology Consortium. Nat. Genet..

[61] Kanehisa M, Goto S (2000). KEGG: kyoto encyclopedia of genes and genomes. Nucleic Acids Res..

[62] Mitchell AL (2019). InterPro in 2019: improving coverage, classification and access to protein sequence annotations. Nucleic Acids Res..

[63] Benson G (1999). Tandem repeats finder: a program to analyze DNA sequences. Nucleic Acids Res..

[64] Xu Z, Wang H (2007). LTR_FINDER: an efficient tool for the prediction of full-length LTR retrotransposons. Nucleic Acids Res..

[65] Tarailo-Graovac, M. & Chen, N. Using RepeatMasker to identify repetitive elements in genomic sequences. *Curr. Protoc. Bioinform.* Chapter 4 Unit 4 **10** (2009). 10.1002/0471250953.bi0410s25 19274634

[66] Bao W, Kojima KK, Kohany O (2015). Repbase Update, a database of repetitive elements in eukaryotic genomes. Mob. DNA.

[67] Delcher AL, Phillippy A, Carlton J, Salzberg SL (2002). Fast algorithms for large-scale genome alignment and comparison. Nucleic Acids Res..

[68] McLaren W (2016). The Ensembl Variant Effect Predictor. Genome Biol..

[69] Wang Y (2012). MCScanX: a toolkit for detection and evolutionary analysis of gene synteny and collinearity. Nucleic Acids Res..

[70] Chiang C (2015). SpeedSeq: ultra-fast personal genome analysis and interpretation. Nat. Methods.

[71] Li H, Durbin R (2009). Fast and accurate short read alignment with Burrows-Wheeler transform. Bioinformatics.

[72] Li H (2009). The Sequence Alignment/Map format and SAMtools. Bioinformatics.

[73] Yourshaw M, Taylor SP, Rao AR, Martin MG, Nelson SF (2015). Rich annotation of DNA sequencing variants by leveraging the Ensembl Variant Effect Predictor with plugins. Brief. Bioinform..

[74] Cockerham CC, Weir BS (1984). Covariances of relatives stemming from a population undergoing mixed self and random mating. Biometrics.

[75] Bolger AM, Lohse M, Usadel B (2014). Trimmomatic: a flexible trimmer for Illumina sequence data. Bioinformatics.

[76] Kim D, Paggi JM, Park C, Bennett C, Salzberg SL (2019). Graph-based genome alignment and genotyping with HISAT2 and HISAT-genotype. Nat. Biotechnol..

[77] Anders S, Pyl PT, Huber W (2015). HTSeq—a Python framework to work with high-throughput sequencing data. Bioinformatics.

[78] Love MI, Huber W, Anders S (2014). Moderated estimation of fold change and dispersion for RNA-seq data with DESeq2. Genome Biol..

[79] Zhou Y (2019). Metascape provides a biologist-oriented resource for the analysis of systems-level datasets. Nat. Commun..

[80] Kumar S, Stecher G, Li M, Knyaz C, Tamura K (2018). MEGA X: molecular evolutionary genetics analysis across computing platforms. Mol. Biol. Evolut..

[81] Robert X, Gouet P (2014). Deciphering key features in protein structures with the new ENDscript server. Nucleic Acids Res..

[82] Heinz S (2010). Simple combinations of lineage-determining transcription factors prime cis-regulatory elements required for macrophage and B cell identities. Mol. Cell.

[83] Tarbell ED, Liu T (2019). HMMRATAC: a Hidden Markov ModeleR for ATAC-seq. Nucleic Acids Res..

[84] Siersbaek R, Mandrup S (2011). Transcriptional networks controlling adipocyte differentiation. Cold Spring Harb. Symp. Quant. Biol..

[85] Sarantopoulos CN (2018). Elucidating the preadipocyte and its role in adipocyte formation: a comprehensive review. Stem Cell Rev. Rep..

[86] Mota de Sa P, Richard AJ, Hang H, Stephens JM (2017). Transcriptional regulation of adipogenesis. Compr. Physiol..

[87] Matsubara Y, Sato K, Ishii H, Akiba Y (2005). Changes in mRNA expression of regulatory factors involved in adipocyte differentiation during fatty acid induced adipogenesis in chicken. Comp. Biochem. Physiol. A Mol. Integr. Physiol..

[88] Wang W (2017). Immortalization of chicken preadipocytes by retroviral transduction of chicken TERT and TR. PloS ONE.

[89] Shang Z (2014). Oleate promotes differentiation of chicken primary preadipocytes in vitro. Biosci. Rep..

[90] Ramirez-Zacarias JL, Castro-Munozledo F, Kuri-Harcuch W (1992). Quantitation of adipose conversion and triglycerides by staining intracytoplasmic lipids with Oil red O. Histochemistry.

[91] Livak KJ, Schmittgen TD (2001). Analysis of relative gene expression data using real-time quantitative PCR and the 2(-Delta Delta C(T)) Method. Methods.

[92] Wang Z (2019). Dynamics of transcriptome changes during subcutaneous preadipocyte differentiation in ducks. BMC Genomics.

