# Supplementary material for: Three chromosome-level duck genome assemblies provide insights into genomic variation during domestication
Source: Nat Commun. 2021 Oct 11;12:5932. doi: 10.1038/s41467-021-26272-1 (PMC8505442; doi:10.1038/s41467-021-26272-1)
Supplement: Supplementary file 1 — Supplementary information [file 41467_2021_26272_MOESM1_ESM.pdf]

Supplementary Information for

Zhu, et al. Three chromosome-level duck genome assemblies provide insights into genomic variation during domestication

Supplementary Figures

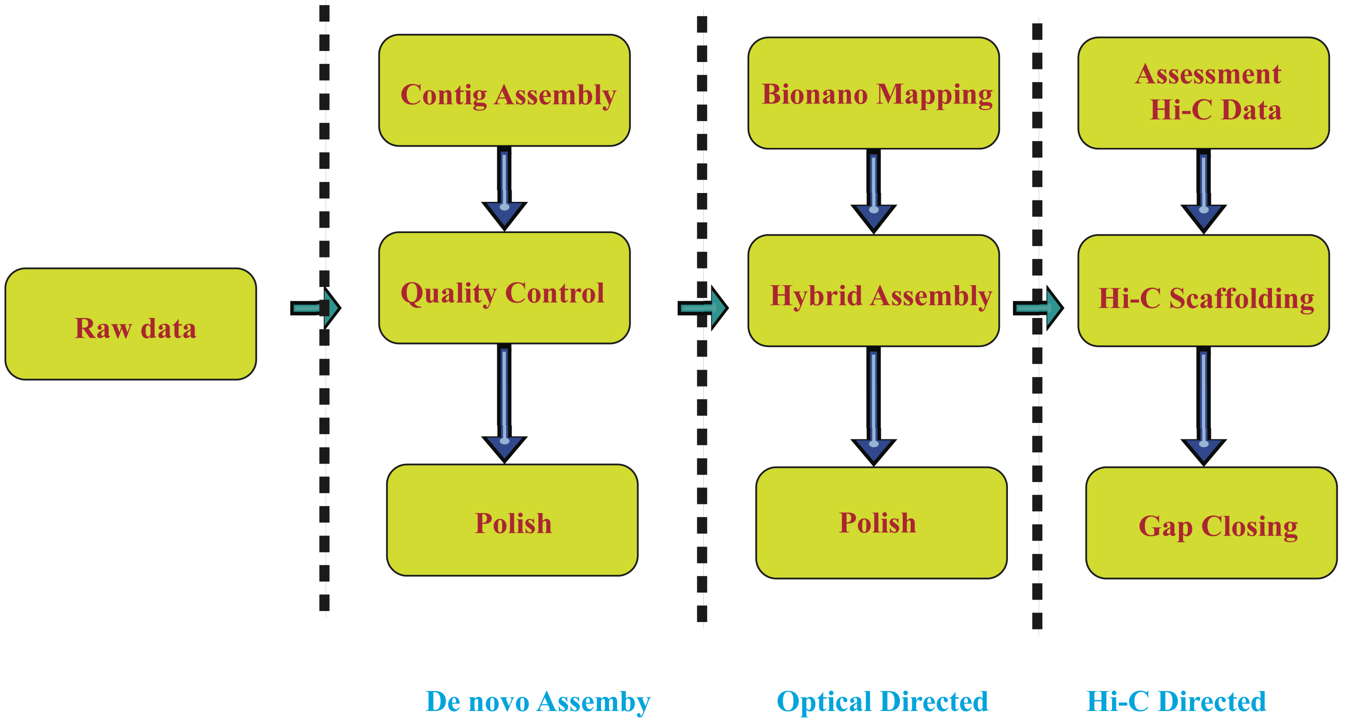

Supplementary Figure 1. The pipeline for multi-level chromosome assembly. Falcon was used for constructing initial contigs. The initial polishing was performed with Quiver using PacBio-only long reads, and then Pilon. Hybrid scaffolding of the PacBio-corrected contigs and the BioNano-based consensus map was performed using the hybrid scaffolding module within IrysView software. After scaffolding, PBJelly from PBSuite was performed to close gaps in the hybrid assembly. The Hi-C sequencing data were first aligned to the assembled contigs/scaffolds using the Bowtie end-to-end algorithm, and then the assembled scaffolds were clustered, ordered, and directed into pseudochromosomes using Lachesis. The final draft was corrected using LR_Gapcloser.

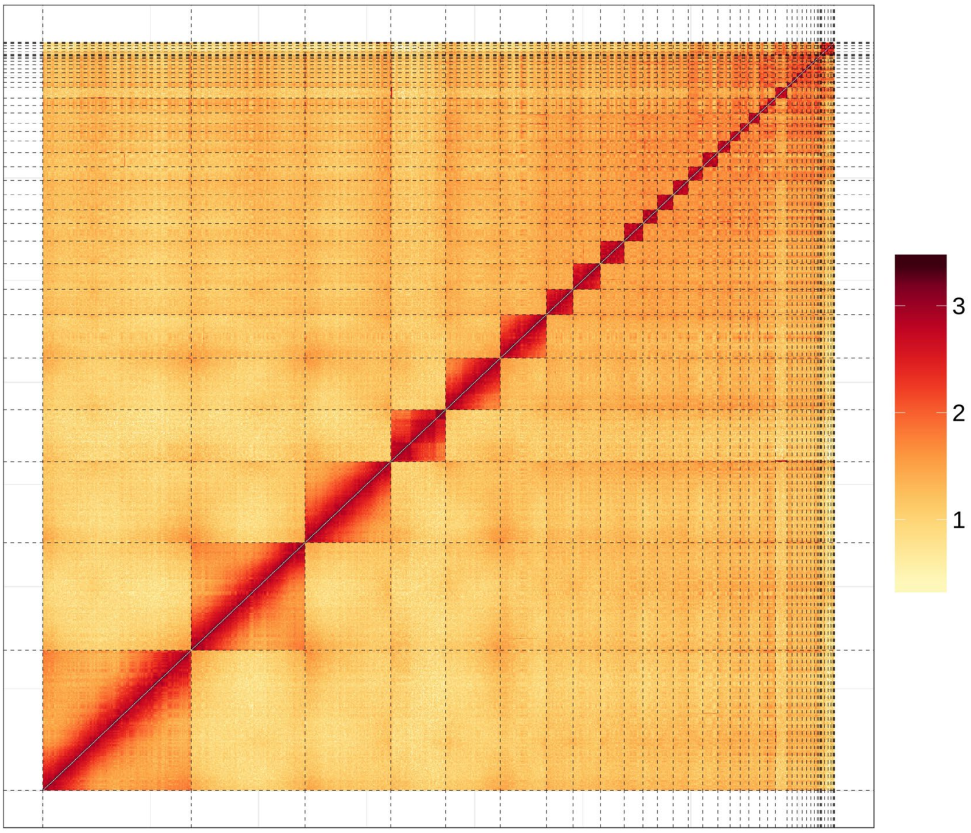

Supplementary Figure 2. Chromatin interactions in each chromosome for Mallard. The Hi-C data in Mallard were mapped to the CAU_wild_1.0 genome. Heatmap is shown at a resolution of 200 Kb. The dark red dots show a high probability of interaction, and light yellow show a low probability of interaction.

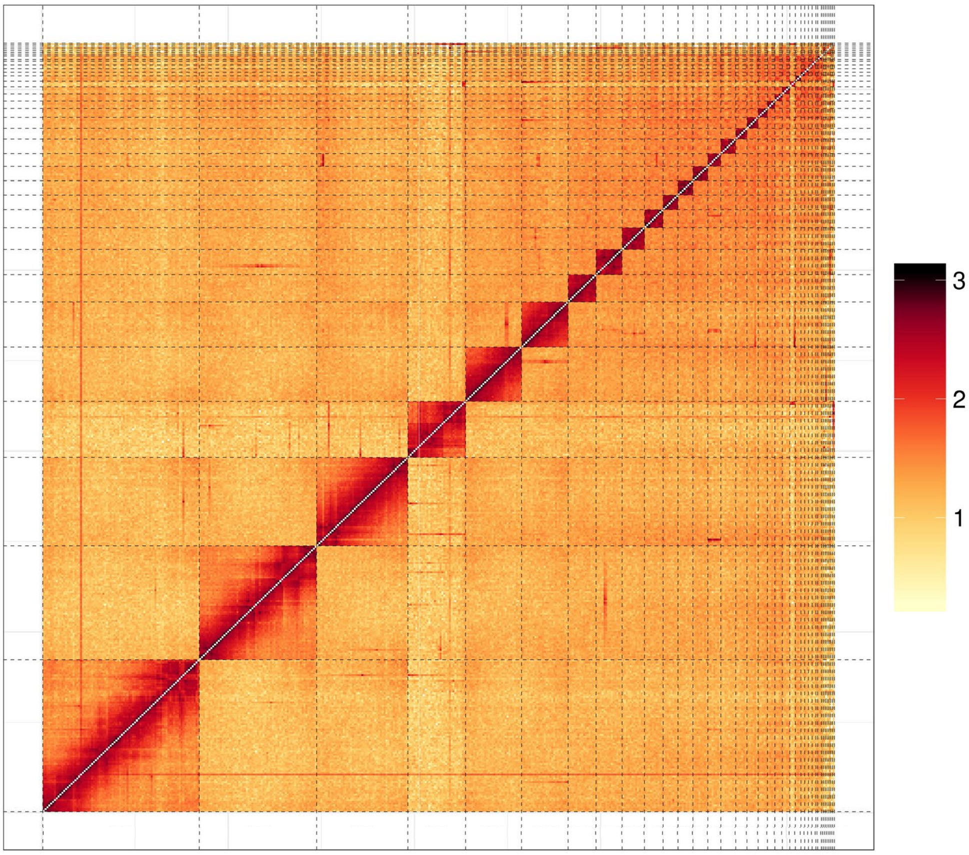

Supplementary Figure 3. Chromatin interactions in each chromosome for Shaoxing duck. The Hi-C data in Shaoxing ducks were mapped to the CAU_laying_1.0 genome. Heatmap is shown at a resolution of 200 Kb. The dark red dots show a high probability of interaction, and light yellow show a low probability of interaction.

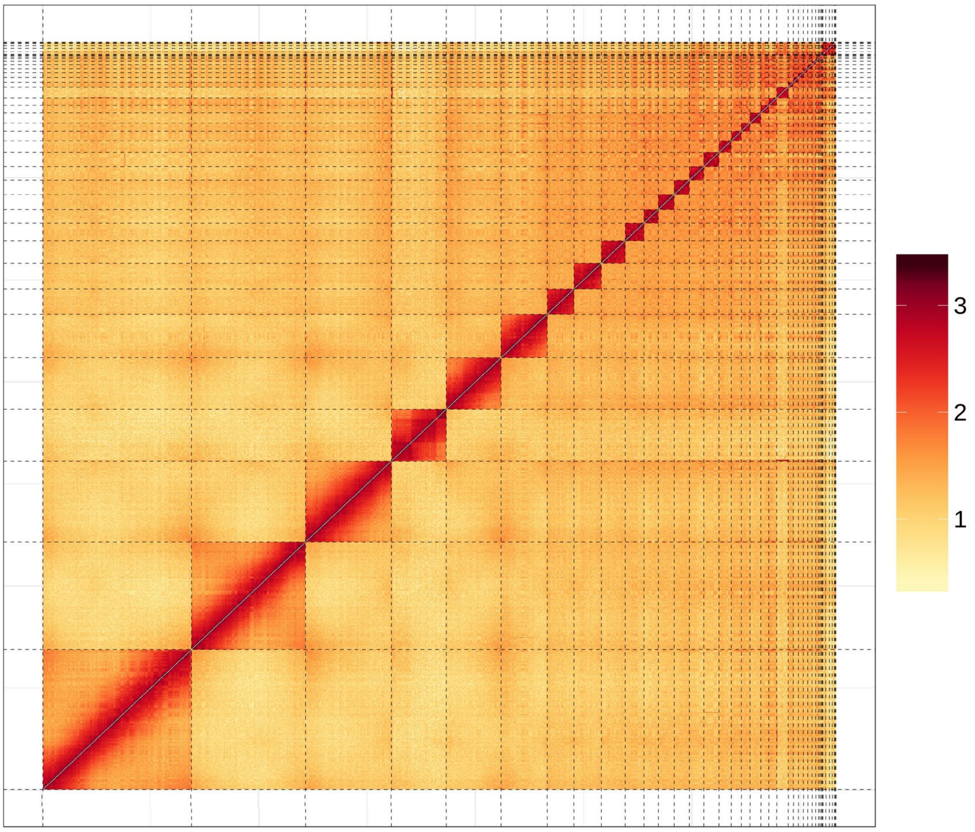

Supplementary Figure 4. Chromatin interactions in each chromosome for Pekin duck. The Hi-C data in Pekin duck were mapped to the CAU_duck_2.0 genome. Heatmap is shown at a resolution of 200 Kb. The dark red dots show a high probability of interaction, and light yellow show a low probability of interaction.

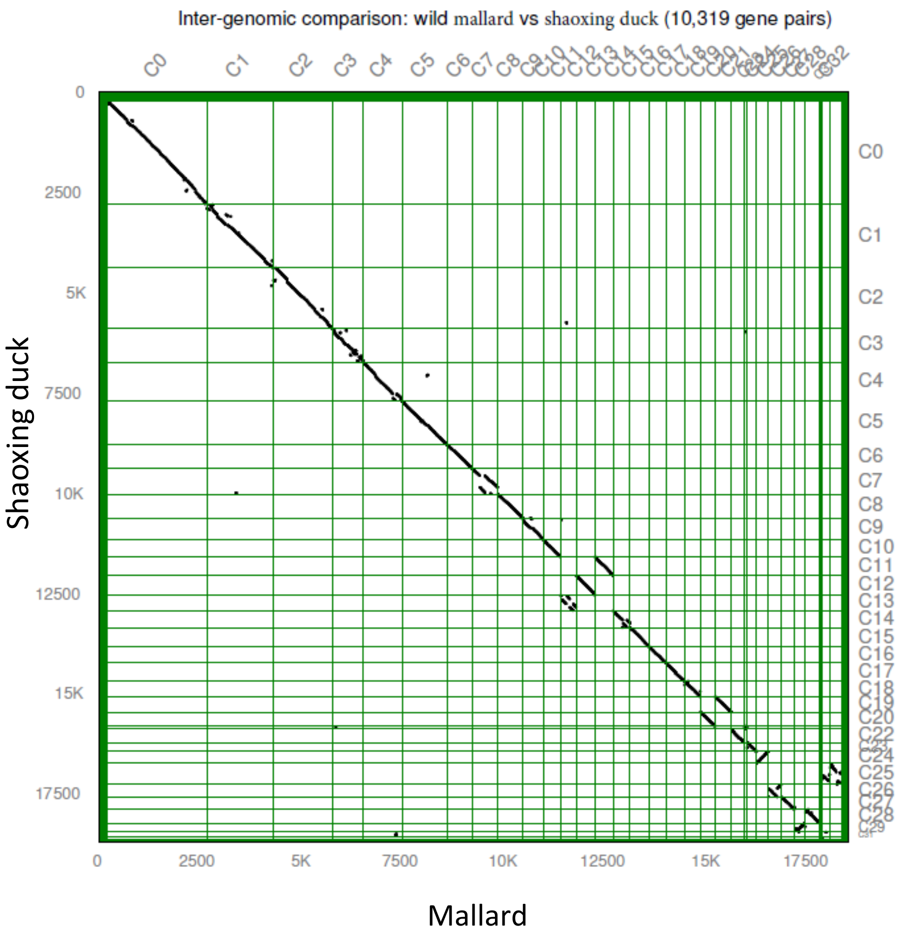

Supplementary Figure 5. Gene collinearity between Mallard and Shaoxing duck genomes.

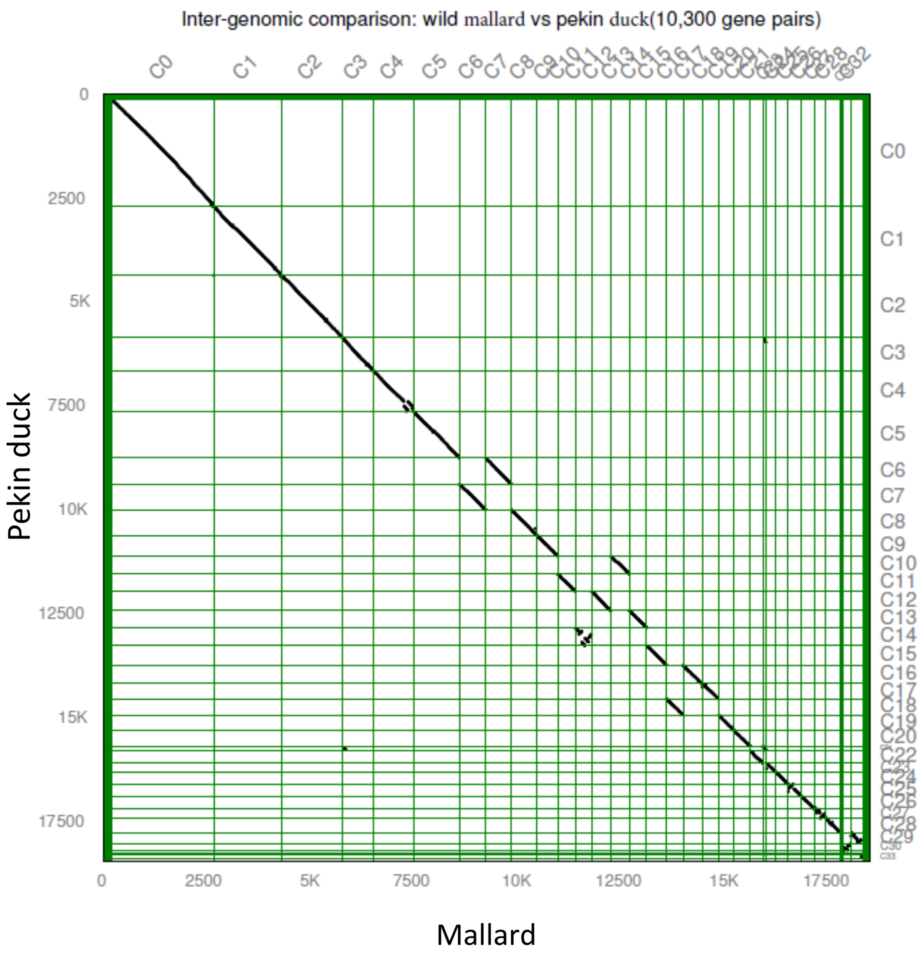

Supplementary Figure 6. Gene collinearity between Mallard and Pekin duck genomes.

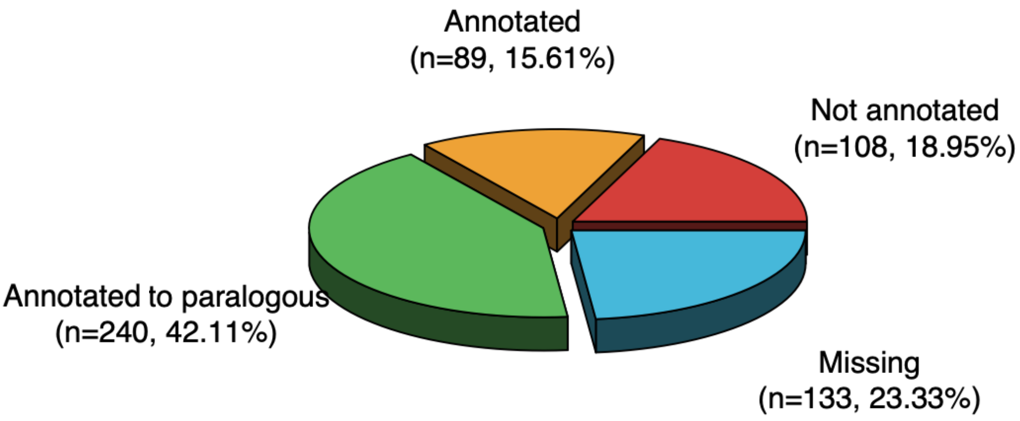

Supplementary Figure 7. Annotation statistics of 'missing genes' for the Mallard genome. Missing genes (89) are annotated in the Mallard genome: 240 genes were annotated as paralogous, 108 genes were not annotated but showed fragment homology, and 133 genes were missing from the Mallard genome.

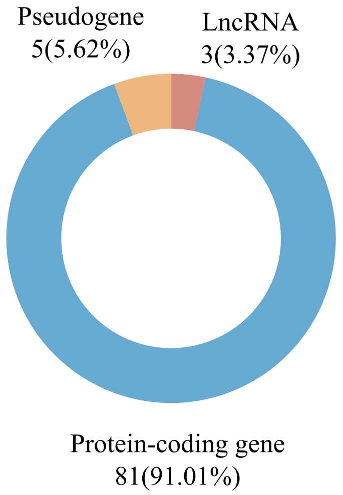

Supplementary Figure 8. Statistics of annotated 'missing gene' types for the Mallard genome. Among the 89 orthologs found in the previously-absent genes, 81 are protein-coding genes, 5 are pseudogenes, and 3 are lncRNA.

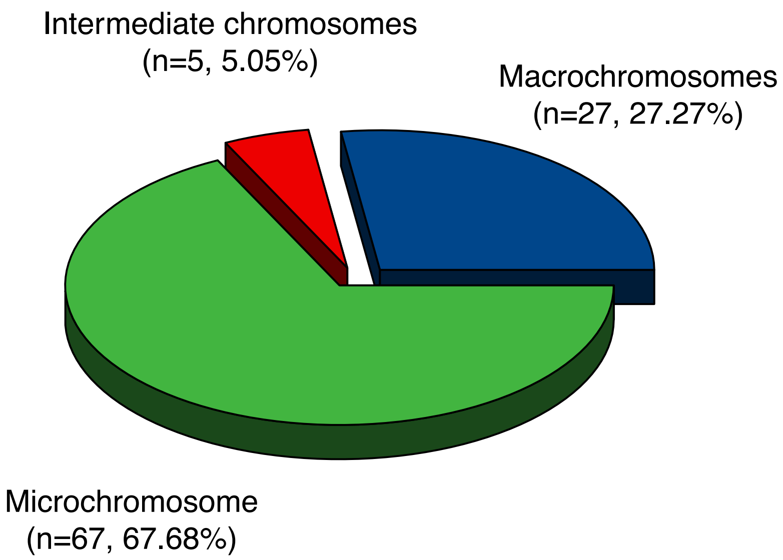

Supplementary Figure 9. The distribution of annotated 'missing gene' locations in the Mallard genome. Among the annotated missing genes, 27 are distributed on the macrochromosomes (chr1-5), 5 on the intermediate chromosomes (chr6-14), and 67 on the microchromosomes (chr15-40 and other super-scaffolds).

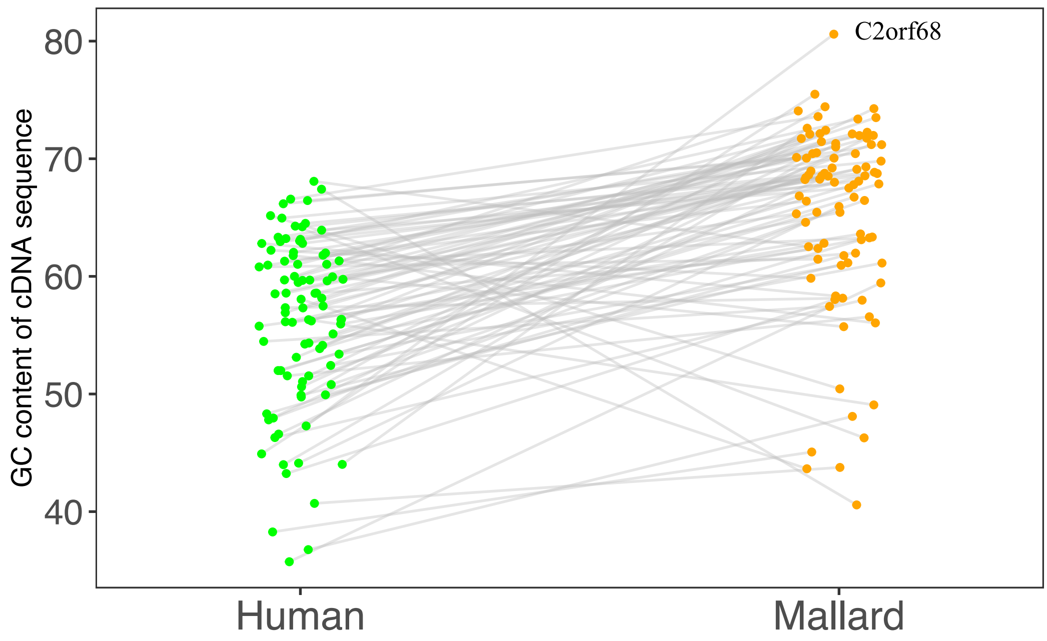

Supplementary Figure 10. Human and Mallard have differentiated GC content for 89 annotated 'missing genes'. Points show the GC content for each gene in Human and Mallard. Gray lines connect GC content between genes, with the slope representing the magnitude of the difference.

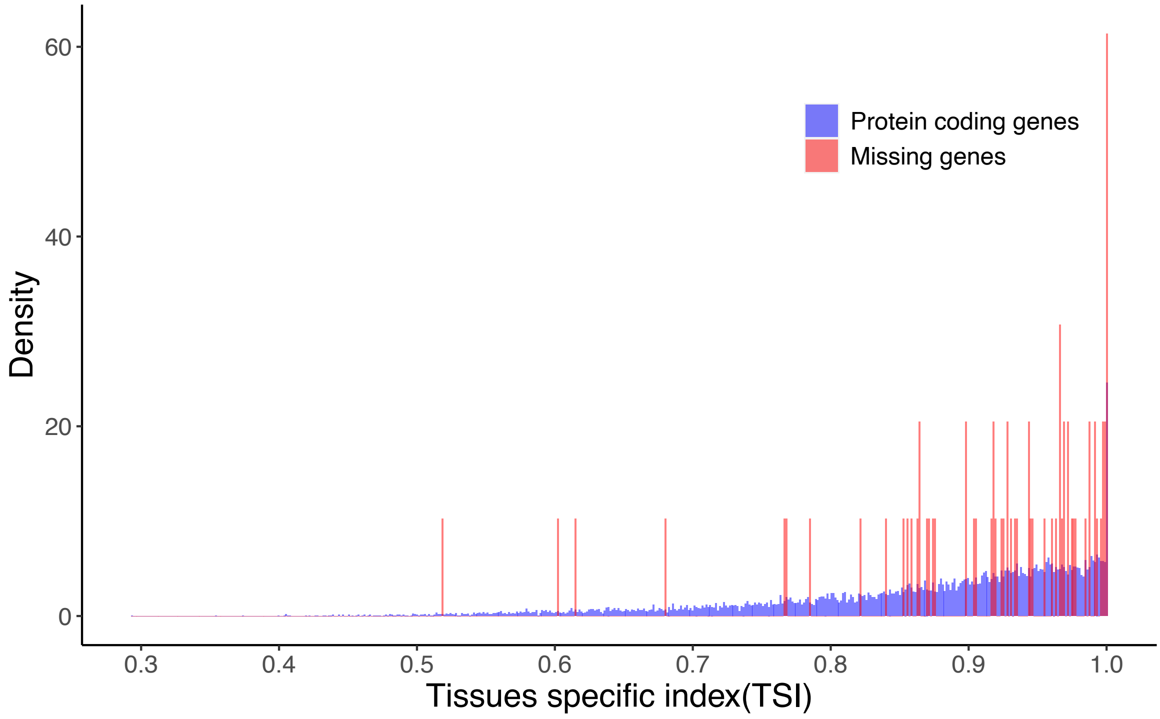

Supplementary Figure 11. Expression pattern of annotated "missing" genes and protein coding genes. Tissue-specific expression index (TSI) of missing genes in duck. TSI of missing genes is significantly higher than the genome background.

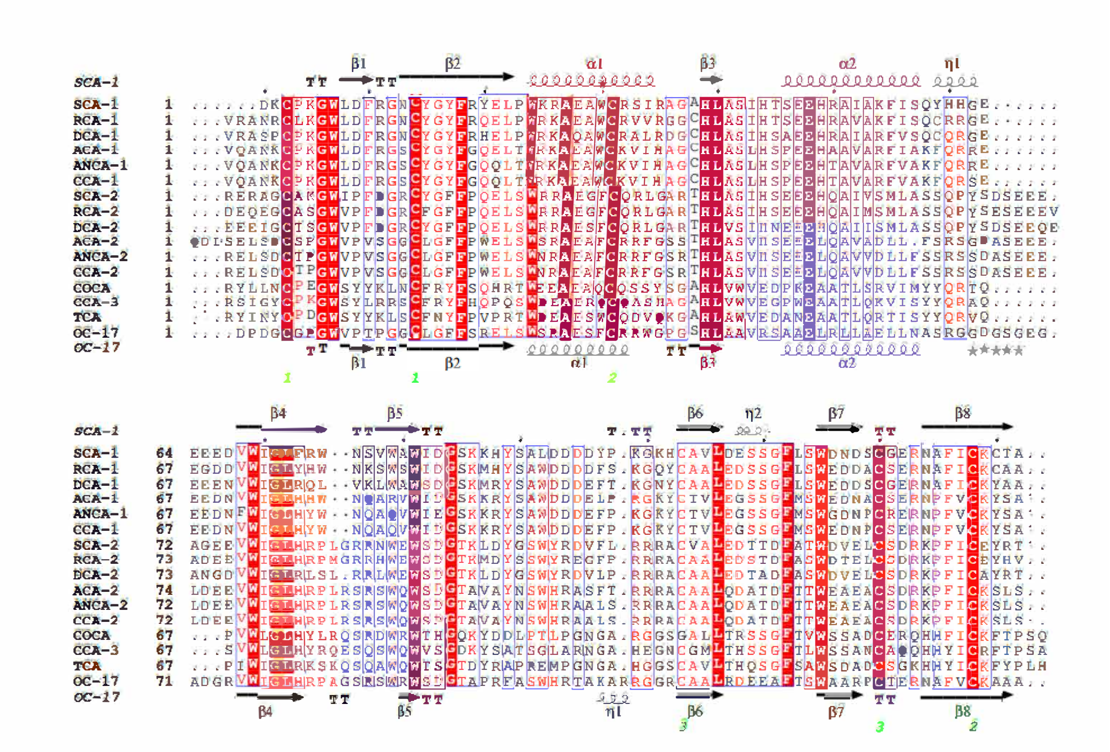

Supplementary Figure 12. Conserved and structural characteristics of the aligned amino acid sequences of OC-17 orthologs in various avian species. Known eggshell CTL proteins of birds were downloaded from the UniProt database, including Ovocleidin-17(OC-17, Q9PRS8), Struthiocalcin-1(SCA-1, P83514), Struthiocalcin-2(SCA-2, P83515), Rheacalcin-1(RCA-1, P84617), Rheacalcin-2(RCA-2, P84618), Dromaiocalcin-1(DCA-1, P84615) and Dromaiocalcin-2(DCA-2, P84616). Multiple alignment was performed using MUSCLE[93] and rendered by ESPript3[81]. The red background indicates complete conservation, and the blue box indicates that the conservation is greater than 50%, and the conserved amino acids are highlighted with red letters. α, β, η indicates that the corresponding amino acids are in α-helix, β-strand, and 310 helical regions; T is the turn. Green numbers indicate cysteine residues and predicted disulfide bond connectivity, while grey asterisks are labeled as alternating conformation.

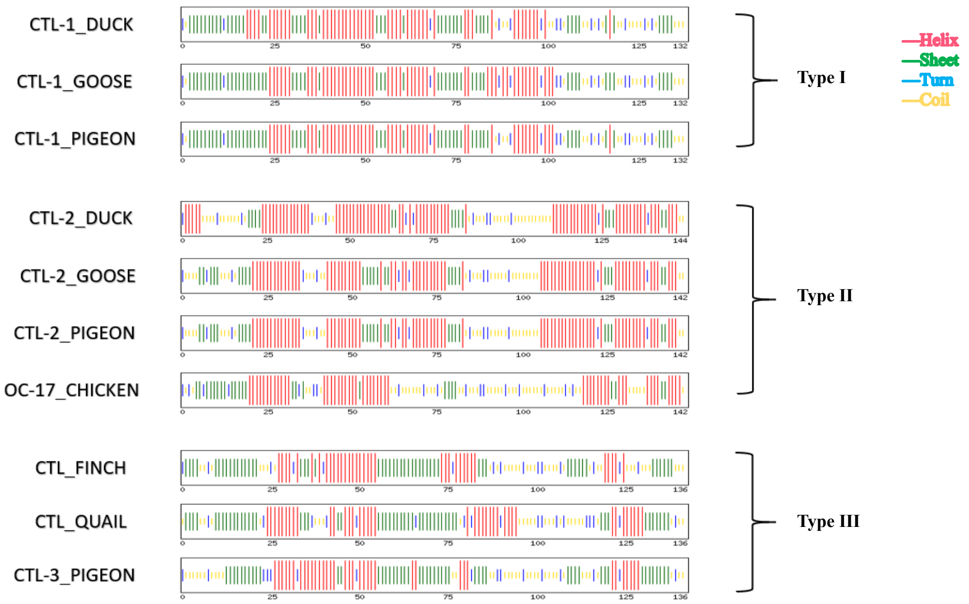

Supplementary Figure 13. Comparative alignments of type I, II, and III eggshell CTL proteins from various bird species. CFSSP was used to predict secondary structure based on three types of CTL proteins. Red line represents alpha helix, green line represents beta sheet, blue line represents turn, and yellow represents random coil.

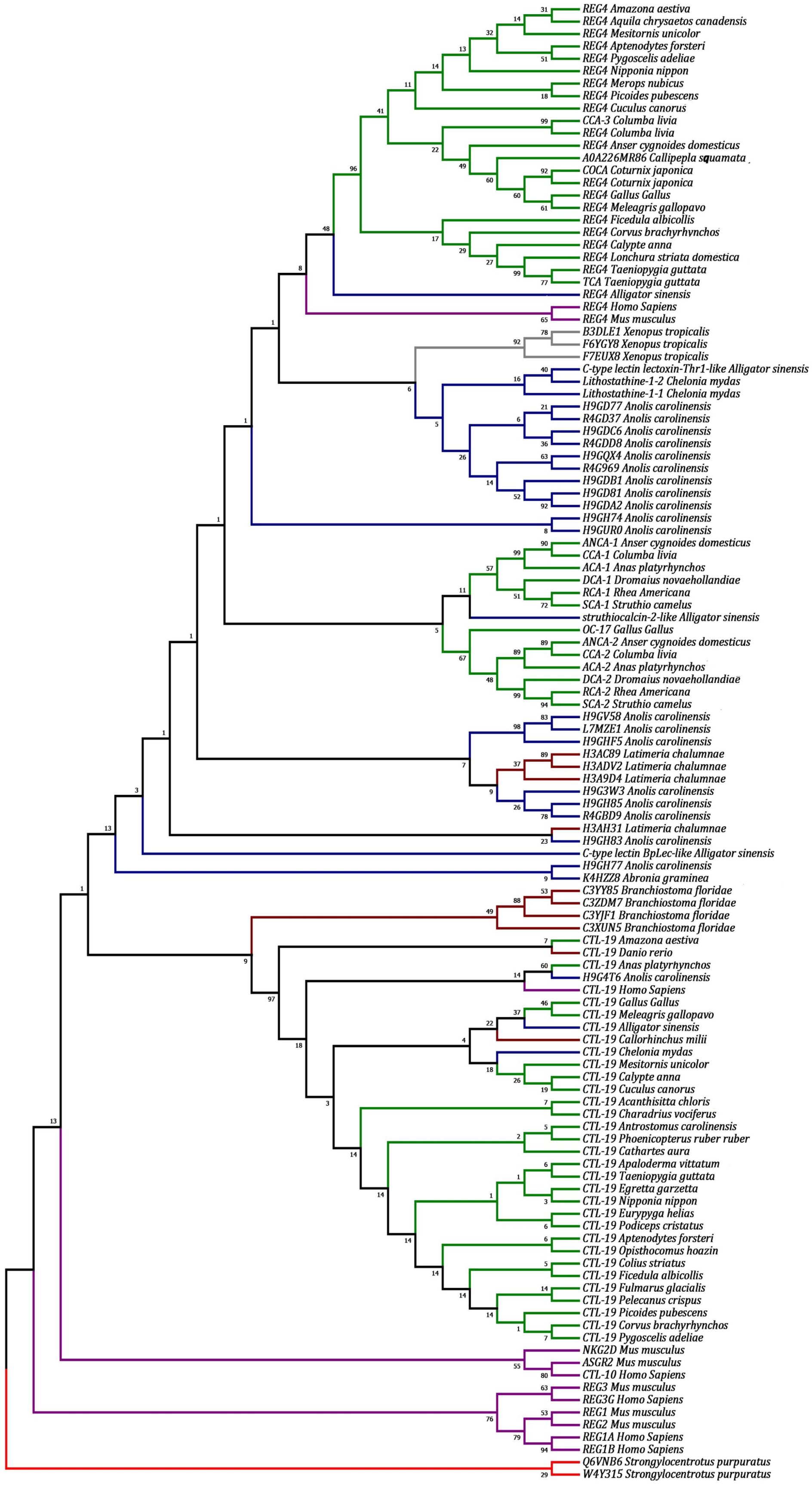

Supplementary Figure 14. Phylogenetic tree for vertebrate CTL members. The 9 CTL proteins of birds mapped to the UniProtKB database. A total of 119 amino acid sequences representing species in different clades (fish, amphibians, reptiles, birds, and mammals) were selected from the top 1000 sequences (E-value < 10^{-10}, Identity > 25%). Taking echinoderm as the outgroup, the phylogenetic tree was established. Red, brown, gray, blue, green, and purple represent echinoderms, fish, amphibians, reptiles, birds, and mammals, respectively. The positions of the orange circles and red squares represent gene duplication and speciation events, respectively.

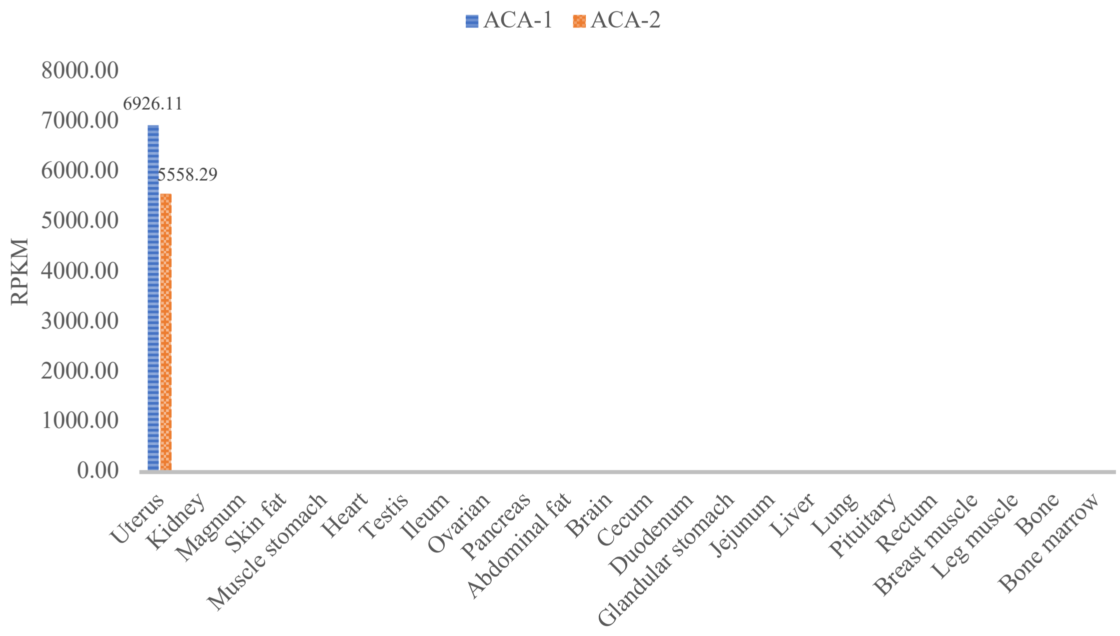

Supplementary Figure 15. ACA-1 and ACA-2 gene expression in different tissues of Pekin duck. The expression levels of ACA-1 and ACA-2 were examined in 24 multi-tissue extracts from duck. The y-axis represents gene expression (RPKM), and the x-axis represents different tissues. In ducks, the expression of ACA-1 and ACA-2 genes were only detected in the uterus.

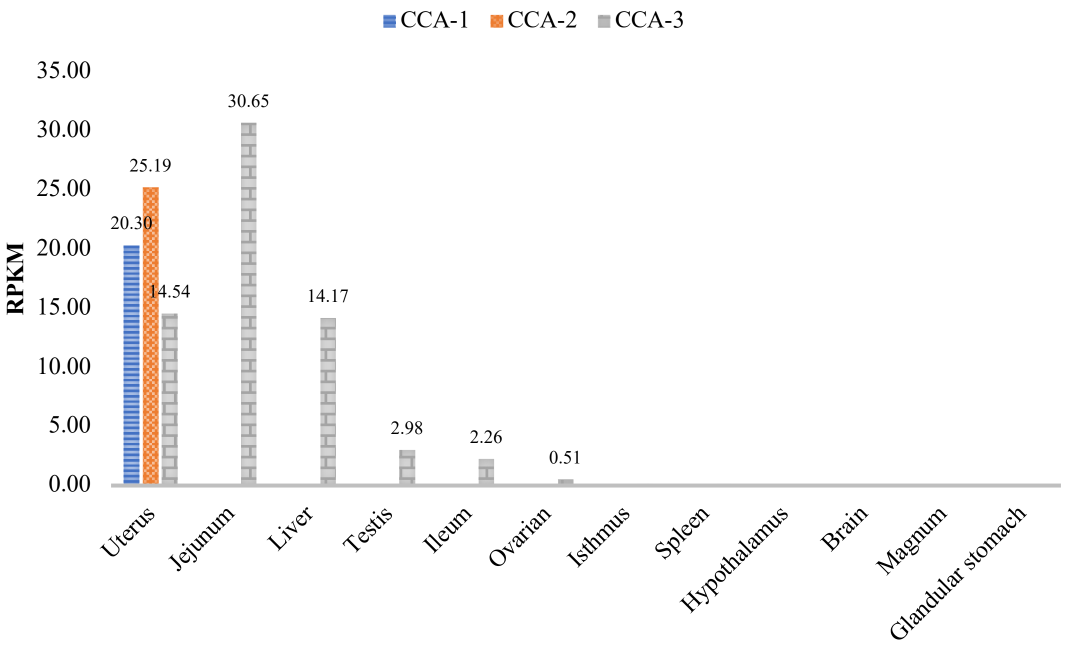

Supplementary Figure 16. CCA-1, CCA-2, and CCA-3 gene expression in different tissues of pigeon. Expression levels of CCA family genes in 12 tissues from pigeon. Among them, CCA-1 and CCA-2 were only expressed in the uterus, while CCA-3 was also expressed in other tissues. Y-axis shows expression levels using RPKM.

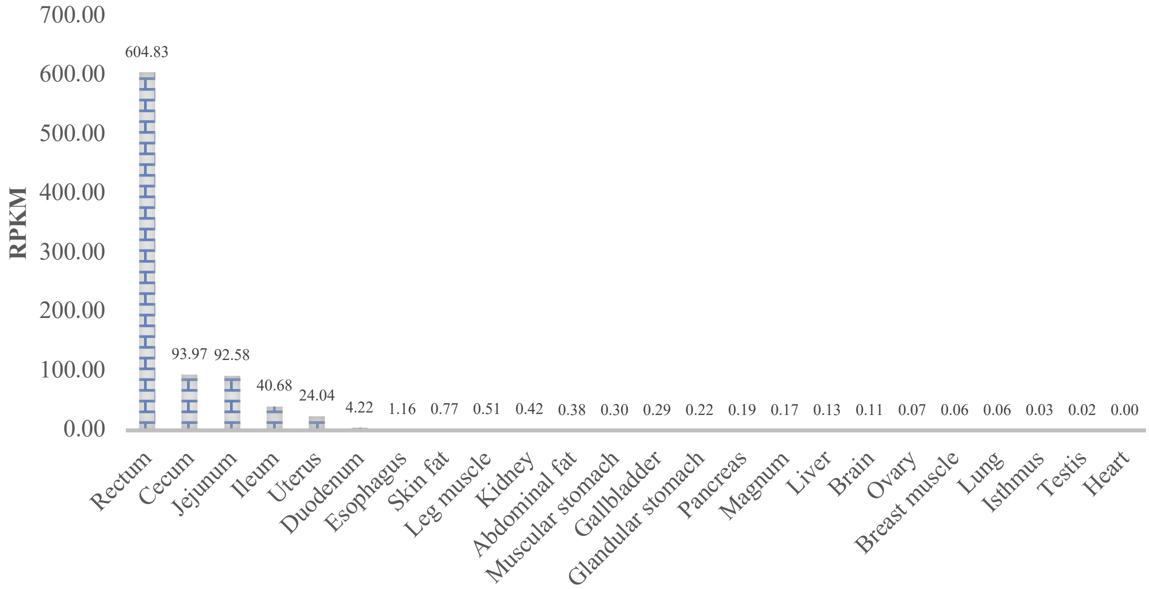

Supplementary Figure 17. TCA gene expression in different tissues of the zebra finch.

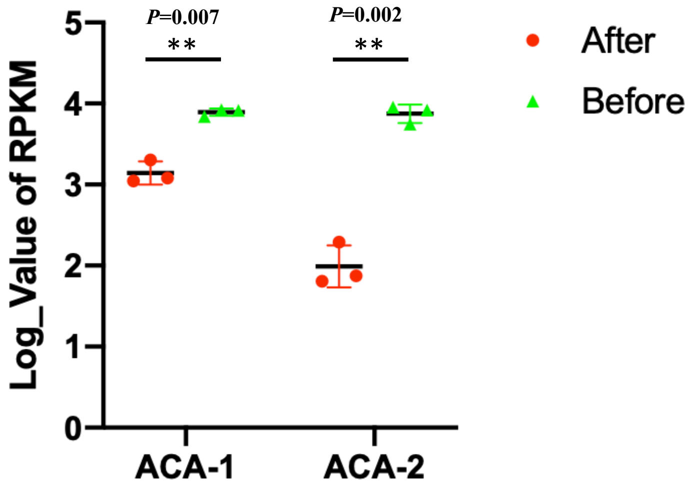

Supplementary Figure 18. Temporal differences in ACA-1 and ACA-2 gene expression in the uterus of Pekin duck. The expression levels of ACA-1 and ACA-2 in the uterus of ducks were significantly different between the active uterus (5-8 h before laying, blue) and the inactive uterus (5-9 h after laying, gray). Statistical significance using two-tailed unpaired Students t-test. Data are represented as mean ± S.D, n =3 biological replicates.

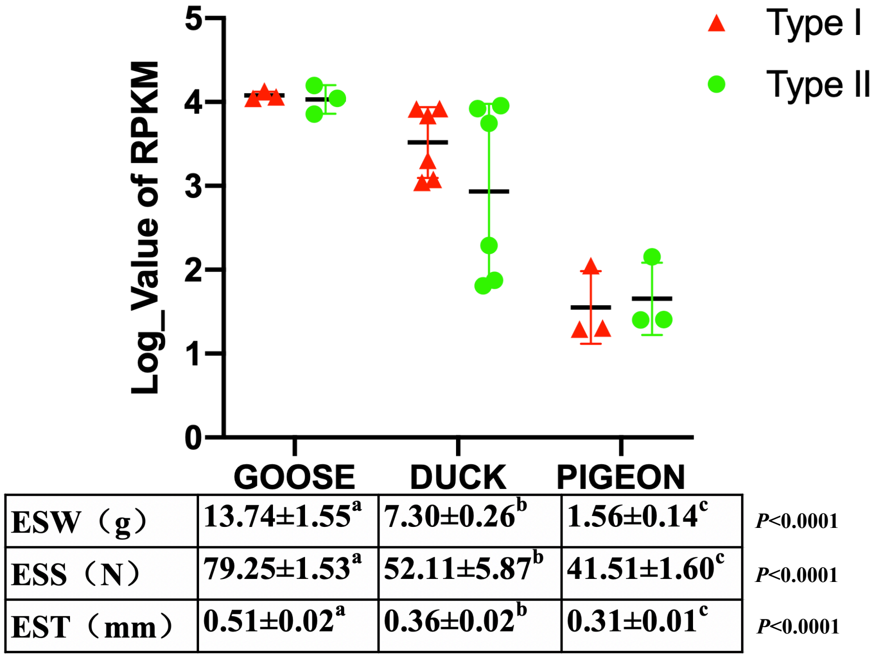

ESW（g）	13.74±1.55[a]	7.30±0.26[b]	1.56±0.14[c]	P<0.0001
ESS（N）	79.25±1.53[a]	52.11±5.87[b]	41.51±1.60[c]	P<0.0001
EST（mm）	0.51±0.02[a]	0.36±0.02[b]	0.31±0.01[c]	P<0.0001

Supplementary Figure 19. Association of CTL gene expression in duck, goose, and pigeon uterus with eggshell quality. Duck, goose, and pigeon type I and type II CTL gene expression levels are correlated with eggshell weight (ESW), eggshell breaking strength (ESS) and eggshell thickness (EST). Different superscripts means significant differences between species in the same row (P<0.05). Statistical significance using two-tailed unpaired Students t-test and one-way analysis of variance. Tukey honestly significant difference was used in multiple comparisons. Data are mean ± S.D, n >=3 biological replicates.

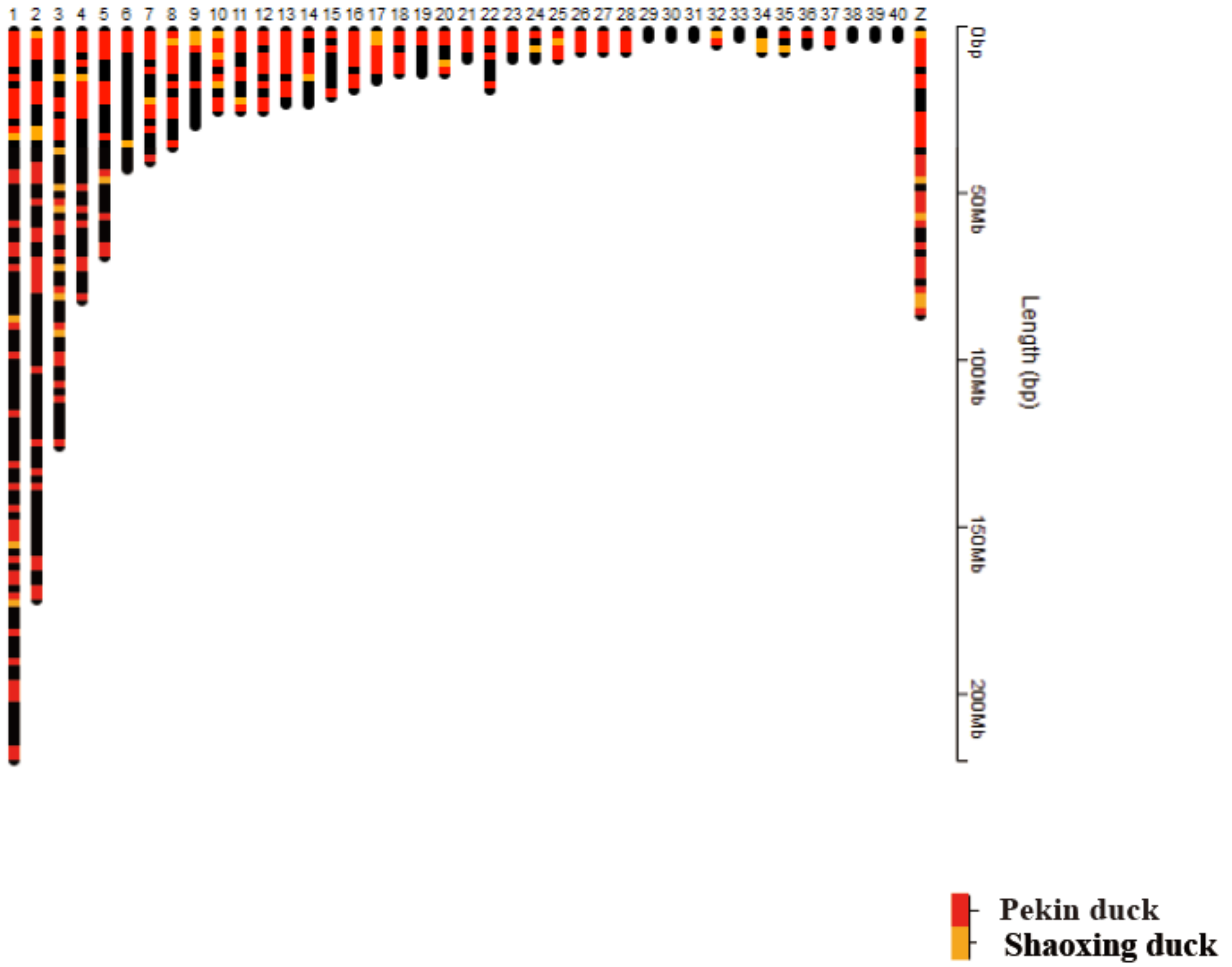

Supplementary Figure 20. The distribution of the presence/absence variations in Mallard genome. Red regions denote the variations between Shaoxing duck and Mallard. Yellow regions denote the variations between Pekin duck and Mallard.

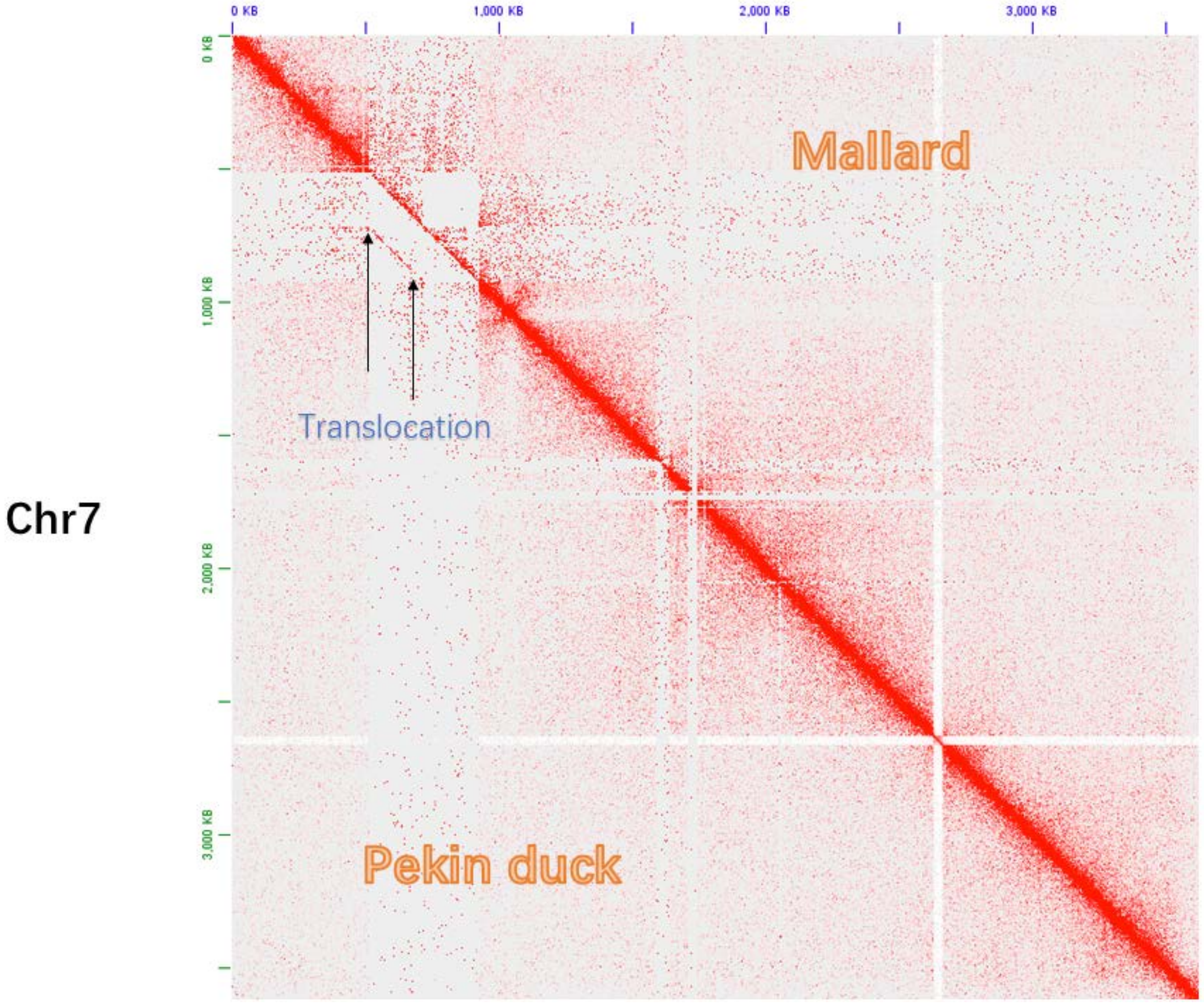

Supplementary table 21. Heat map of chromosome 7 (0-4MB) chromatin contact matrices generated by aligning a Hi-Cdataset of Mallard and Pekin duck to the Mallard genome. The upper triangle is Mallard data, and the lower triangle is Pekin duck data. Hi-C interaction maps indicating a small translocation in the Chr7.

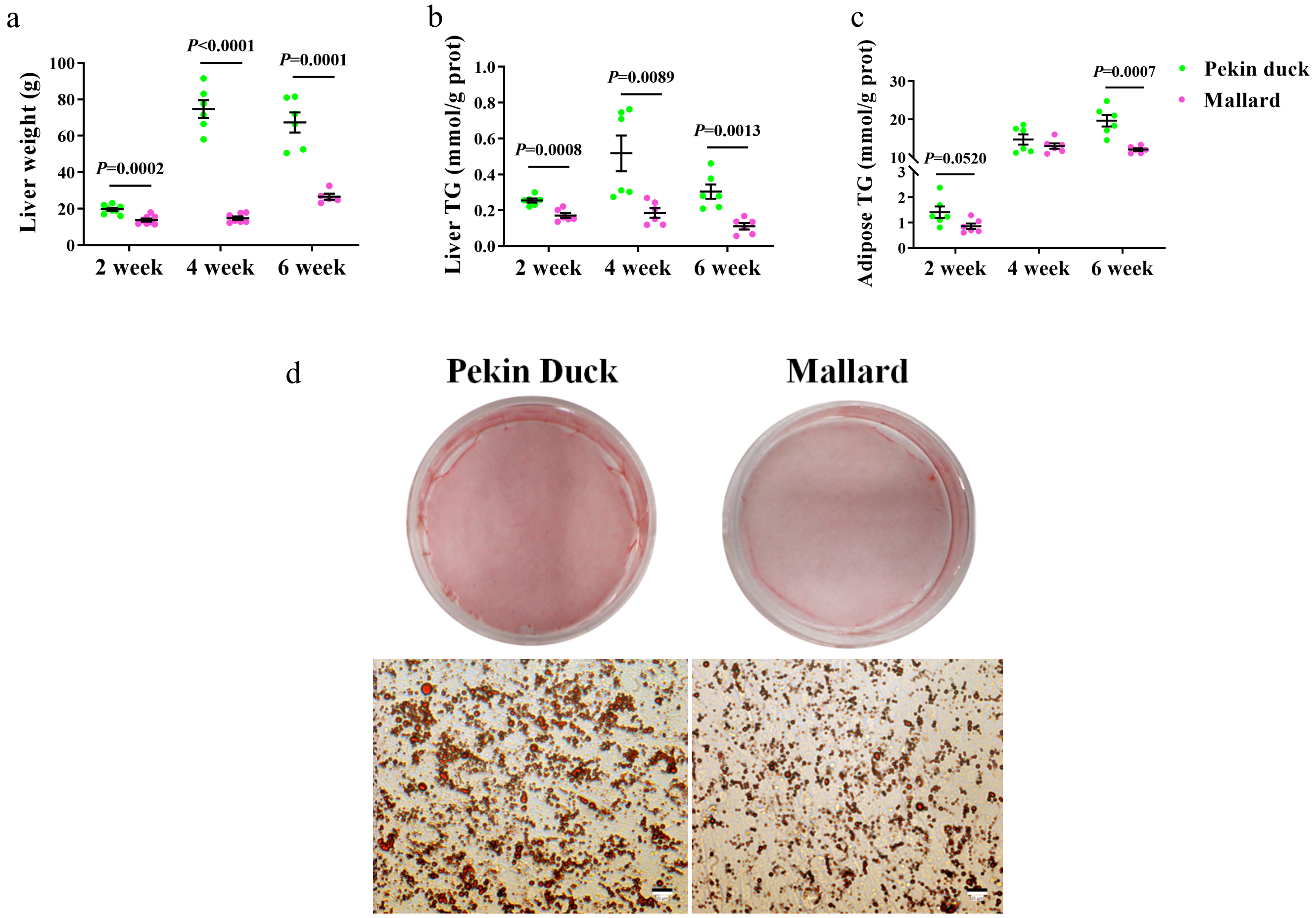

Supplementary Figure 22. Identification of differences in liver and subcutaneous fat deposits between Pekin duck and Mallard. **a** Liver weight in Pekin duck and Mallard at age of 2, 4, 6 weeks (n = 8 Pekin duck, n = 8 Mallard at 2 weeks; n = 6 Pekin duck, n = 7 Mallard at 4 weeks; n = 6 Pekin duck, n = 5 Mallard at 6 weeks). **b, c** Triglyceride content analysis for the hepatic and subcutaneous fat tissue in Pekin duck and Mallard at age of 2, 4, 6 weeks. The Triglyceride concentration assay was used to measure the triglyceride content. *n* = 6 biological replicates. **d** Oil Red O staining to assess lipid accumulation of Pekin duck and Mallard subcutaneous preadipocytes at day 7 post induction. The scale bar represents 200 μm. n = 3 biological replicates. Data are presented as mean ± SEM. Statistical significance was tested using the two-tailed unpaired Student's t-test for **a**, **b** and **c**.

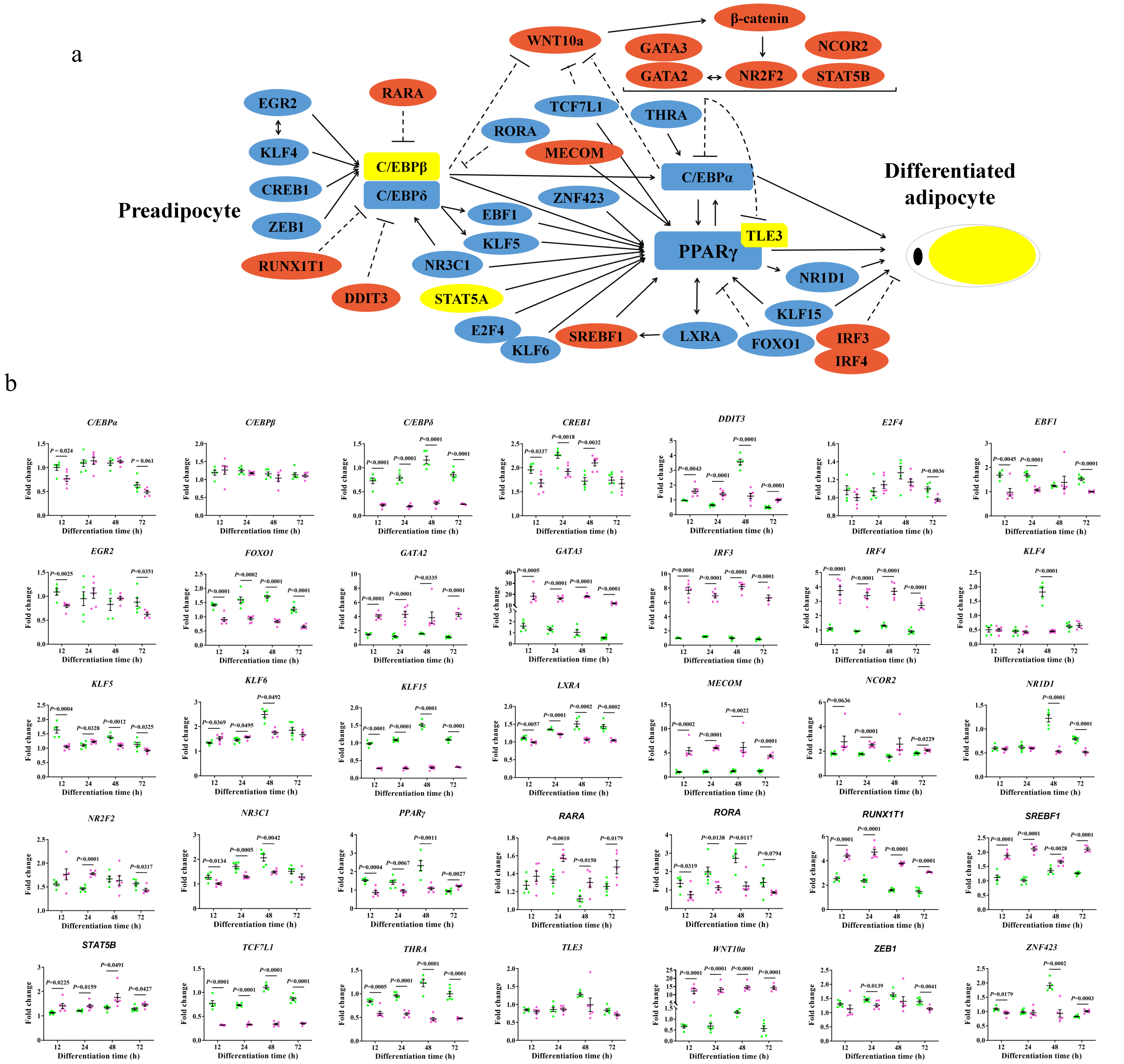

Supplementary Figure 23. Expression comparison of the key transcription factors known to be involved in adipogenesis (Pekin duck and Mallard). a Outline of the transcriptional network controlling preadipocyte differentiation. The network was assembled on the 'Adipogenesis' Pathway scaffold from WikiPathways, incorporating reviews and recent publications of novel adipogenic regulators. Solid lines indicate the activation of gene expression, dashed lines indicate the inhibition of gene expression, and solid lines with arrowheads at both ends indicate that transcription factors are found in the same complex. Genes in blue were highly expressed in Pekin duck. Genes in red were highly expressed in Mallard. Genes in yellow were not differentially expressed between the two duck breeds. **b** Fold changes of adipogenic transcription factors in subcutaneous preadipocytes at 12 h, 24 h, 48 h, 72 h post induction compared to 0 h, as measured by RNA-seq, for Pekin duck (green) and Mallard (pink). RNA-Seq analysis, n =5, subcutaneous preadipocytes of Pekin duck at 0 h, 12 h, 48 h post induction; n = 6, subcutaneous preadipocytes of Pekin duck at 24 h, 72 h post induction; n = 6, subcutaneous preadipocytes of Mallard at 0 h, 12 h, 24 h, 48 h post induction; n = 5, subcutaneous preadipocytes of Mallard at 72 h post induction. Data are presented as mean ± SEM. Statistical significance was tested using the two-tailed unpaired Student's t-test for **b**.

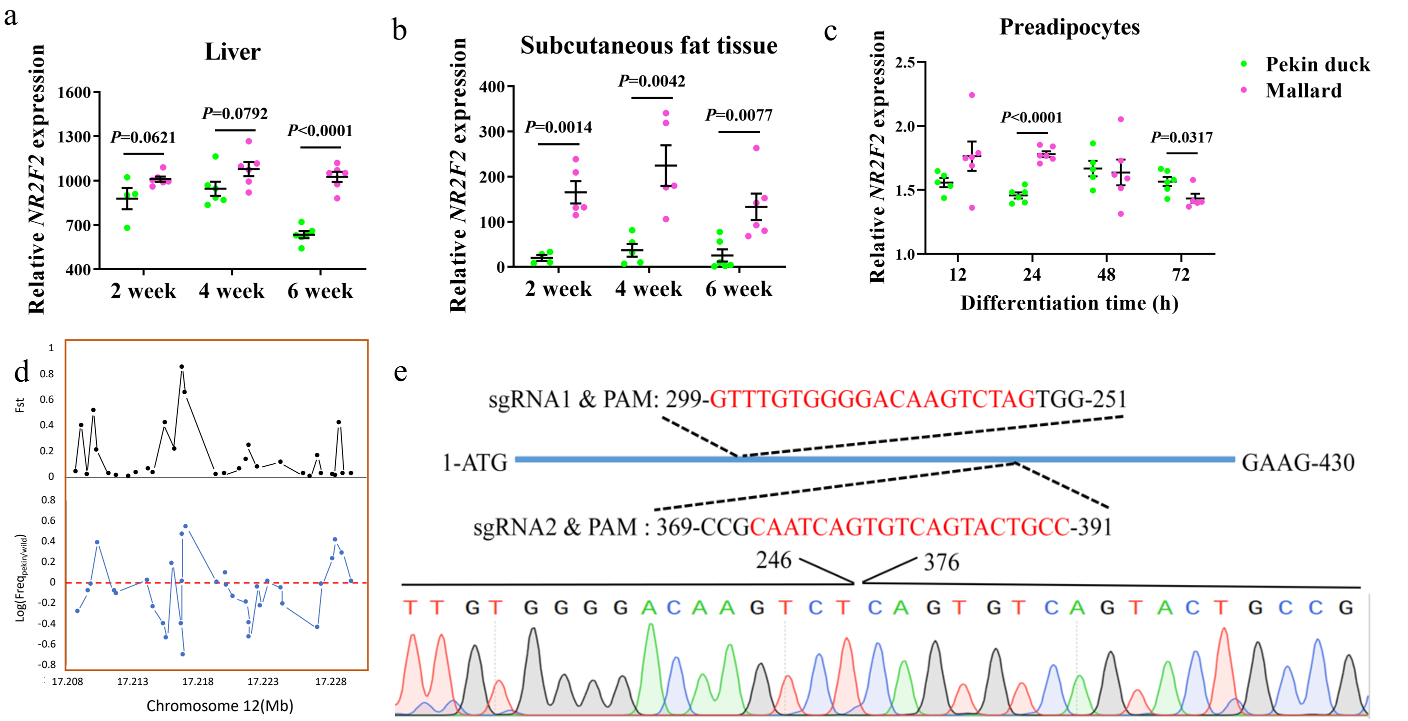

Supplementary Figure 24. ***NR2F2*** **gene expression levels in Pekin duck and Mallard tissues. a** RNA-seq analysis of *NR2F2* expression in liver of Pekin duck and Mallard at age of 2, 4, 6 weeks ($n = 4$ in Pekin duck at the age of 2 weeks; $n = 6$ in Pekin duck at age of 4, 6 weeks; $n = 6$ in Mallard at age of 2, 4, 6 weeks). **b** RNA-seq analysis of *NR2F2* expression in subcutaneous fat tissue of Pekin duck and Mallard at age of 2, 4, 6 weeks (RNA-seq analysis, $n = 4$ in Pekin duck at age of 2 weeks; $n = 5$ in Pekin duck at age of 4 weeks; n=6 in Pekin duck at age of 6 weeks; $n = 5$ in Mallard at age of 2, 4 weeks; $n = 6$ in Mallard at age of 6 weeks). **c** Fold changes of *NR2F2* in preadipocytes at 12 h, 24 h, 48 h, 72 h post induction compared to 0 h, as measured by RNA-seq (RNA-seq analysis, $n = 5$, subcutaneous preadipocytes of Pekin duck at 0 h, 12 h, 48 h post induction; $n = 6$, subcutaneous preadipocytes of Pekin duck at 24 h, 72 h post induction; $n = 6$, subcutaneous preadipocytes of Mallard at 0 h, 12 h, 24 h, 48 h post induction; $n = 5$, subcutaneous preadipocytes of Mallard at 72 h post induction). **d**. The distribution of SNPs with a different frequency in *NR2F2* for Pekin duck and Mallard populations. **e** Schematic diagram of *NR2F2* partial protein coding region and the two targeting loci for *NR2F2* sgRNA (red). DNA sequence map around the targeted locus of the deleted region from *NR2F2*^{KO} clone with both gRNAs. Data are presented as mean ± SEM. Statistical significance was tested using the two-tailed unpaired Student's t-test for **a**, **b** and **c**.

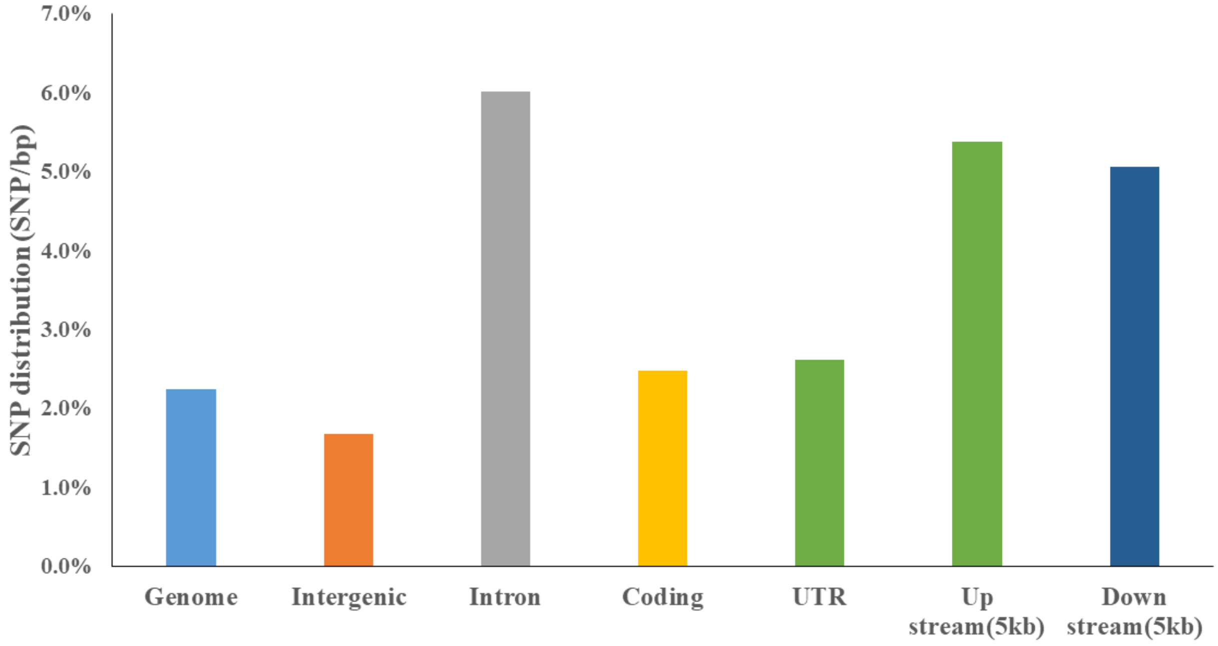

Supplementary Figure 25. The SNP distribution in the different genomic regions.

Supplementary Tables

Supplementary Table 1: Summary of PacBio reads for wild and domestic Mallard ducks. and reads after filtering . These represent a summary of raw sequence data generated from PacBio Sequel RSII

Species	Filter	Total read bases (bp)	Read number	Read N50	Mean read length
Mallard	before filtering	112,261,231,468	10,767,626	16,824	10,425
	after filtering	74,402,479,629	3,535,352	21,073	21,045
Pekin Duck	before filtering	114,832,622,236	10,461,088	17,256	10,977
	after filtering	74,404,037,206	3,421,089	21,590	21,749
Shaoxing Duck	before filtering	112,733,154,740	8,390,380	19,731	13,436
	after filtering	73,959,001,629	3,005,300	24,658	24,610

Supplementary Table 2: Summary of subreads for duck genome accessions. These are after further filters to remove short reads and sequencing adapters.

Species	Length (bp)	Number	Total length (bp)	Average length (bp)
Mallard	0-2000	69	80,411	1,165
	2000-4000	88	272,678	3,099
	4000-6000	96	481,283	5,013
	6000-8000	61	431,967	7,081
	8000-10000	77	687,759	8,932
	10000-12000	102	1,094,701	10,732
	12000-14000	58	754,893	13,015
	14000-16000	94	1,412,390	15,025
	16000-18000	47	803,841	17,103
	>18000	1,020	1,213,526,541	1,189,732
	Total	1,712	1,219,546,464	1,270,899
Pekin duck	0-2000	0	0	0
	2000-4000	2	5,303	2,652
	4000-6000	0	0	0
	6000-8000	0	0	0
	8000-10000	4	37,133	9,283
	10000-12000	19	191,765	10,093
	12000-14000	5	64,255	12,851
	14000-16000	6	92,643	15,441
	16000-18000	9	155,416	17,268
	>18000	427	1,192,451,674	2,792,627
	Total	472	1,192,998,189	2,860,214
Shaoxing duck	0-2000	7	6,928	990
	2000-4000	10	27,482	2,748
	4000-6000	5	26,284	5,257
	6000-8000	2	12,631	6,316
	8000-10000	4	34,409	8,602
	10000-12000	3	33,163	11,054
	12000-14000	3	37,732	12,577
	14000-16000	2	30,537	15,269
	16000-18000	1	17,170	17,170
	>18000	260	1,219,079,593	4,688,768
	Total	297	1,219,305,929	4,768,750

Supplementary Table 3. Summary of BioNano data for duck species.

	Mallard	*Pekin duck*	*Shaoxing duck*
BioNano high quality data			
Filtered	150kb	150kb	150kb
Enzyme	BssS1	BssS1	BssS1
Mol N50 (kb)	233	307	420
Lab (/100kb)	12.67	10.64	10.25
BioNano assembly results			
Total Length (Mb)	1,219.45	1,193.18	1,313.35
Mean Length (Mb)	0.52	1.05	0.66
Median Length (Mb)	35.27	11.92	9.33
L50 Length (Mb)	8.46	5.74	4.01
Maximum Length (Mb)	35.27	44.29	45.23

Supplementary Table 4: Summary of genome assemblies for Mallard and domestic ducks.

Statistics	PacBio Assembly	BioNano Scaffolding*	BioNano Scaffolding#	Hi-C Grouping	LR
Mallard					
Contig/Scaffold number	2,517	2,517	2,514/2,336	2661 /1712	1,986/1,712
Contig/Scaffold length	1,208,310,112	1,207,917,279	[illegible]207,805,831/1,219,453,6[illegible]	1,207,805,831/1,219,538,895	[illegible],210,757,988/1,219,546,464
Contig/Scaffold N50 (Mbp)	4.68	4.68	4.76/8.46	4.65/77.63	11.30/77.63
Contig/Scaffold N90 (Mbp)	0.4	0.40	0.40/0.65	0.36/7.21	1.37/7.21
Contig/Scaffold Max (Mbp)	23.38	**23.38**	**23.38/35.27**	23.38/208.32	73.51/208.33
Gap length (bp)	#	**#**	**11,647,864**	11,733,064	8,788,476
**Gap Number**	#	#	181	1,077	391
Pekin duck					
Contig/Scaffold number	2061	2,061	1118 /915	1175 / 472	622/472
Contig/Scaffold length	1,207,786,711	1,207,622,034	[illegible]179,897,621/1,192,925,1[illegible]	1,179,897,621 /1,192,982,887	[illegible],185,139,540/1,192,998,189
Contig/Scaffold N50 (Mbp)	5.46	5.46	5.98/11.92	5.85/76.28	16.04/76.28
Contig/Scaffold N90 (Mbp)	0.44	0.44	0.62/1.25	0.60/7.64	1.80/7.64
Contig/Scaffold Max (Mbp)	21.95	21.95	21.95/44.29	21.94/207.24	16.04/76.28
Gap length (bp)	#	###	13,027,566	13,085,266	7,858,649
Gap Number			203	830	273
Shaoxing duck					
Contig/Scaffold number	3200	2,934/1,989	1,275 /540	1,367/ 297	859/297
Contig/Scaffold length	1288119988	[illegible]01,752,329/1,313,358[illegible]	[illegible]08,135,341/1,219,271,7[illegible]	1,208,135,341/1,219,305,859	[illegible],210,592,931/1,219,305,929
Contig/Scaffold N50 (Mbp)	3.79	4.01/9.33	4.53 /10.45	4.22/76.92	8.56/ 76.92
Contig/Scaffold N90 (Mbp)	0.19	0.22/0.49	0.63/1.56	0.56/7.19	1.09/7.19
Contig/Scaffold Max (Mbp)	29.91	32.08/45.02	32.08/45.02	27.70/212.53	53.93/ 212.53
Gap length (bp)	#	11,606,298	11,136,418	11,170,518	8,712,998
Gap Number		**870**	735	1030	559

*Characterization of contigs after BioNano scaffolding.
#Characterization of scaffolds after BioNano scaffolding

Supplementary Table 5. Summary of Hi-C read mapping.

	Number of Reads	Percentage%
MALLARD		
Total read pairs	3,725,771	
Uniquely mapped read pairs	3,128,156	83.96%
Valid interaction pairs	2,379,245	63.86%
Dangling end pairs	7,877	0.21%
Re-ligation pairs	32,194	0.86%
Self-cycle pairs		
Dumped pairs	118,733	3.19%
Pekin Duck		
Total read pairs	4,430,634	
Uniquely mapped read pairs	3,685,017	83.17%
Valid interaction pairs	2,315,891	52.27%
Dangling end pairs	19,509	0.44%
Re-ligation pairs	138,374	3.12%
Self-cycle pairs		
Dumped pairs	474,671	10.71%
Shaoxing Duck		
Total read pairs	4,290,853	
Uniquely mapped read pairs	3,512,911	81.87%
Valid interaction pairs	2,390,706	55.72%
Dangling end pairs	12,987	0.30%
Re-ligation pairs	106,221	2.48%
Self-cycle pairs		
Dumped pairs	272,424	6.35%

Supplementary Table 6. Summary of the Hi-C grouping results in each pseudo chromosome.

	Mallard(CAU_wild_1.0)		Pekin Duck(CAU_duck_2.0)			Shaoxing Duck(CAU_laying_1.0)		
Chr*	Scaffold number	Total length	Chr	Scaffold number	Total length	Chr	Scaffold number	Total length
Chr1	125	208,324,881	Chr1	67	207,243,756	Chr1	53	212,526,315
Chr2	101	162,939,720	Chr2	38	159,703,539	Chr2	45	161,641,363
Chr3	42	119,723,606	Chr3	21	119,484,232	Chr3	25	123,865,793
Chr4	38	77,626,185	Chr4	15	76,279,536	Chr4	8	76,919,223
Chr5	19	64,988,526	Chr5	11	65,110,970	Chr5	10	64,623,077
Chr6	2	39,543,298	Chr6	18	38,344,719	Chr6	4	39,707,607
Chr7	9	37,812,880	Chr7	2	39,447,265	Chr7	5	38,613,382
Chr8	16	33,351,727	Chr8	7	33,094,545	Chr8	8	32,602,112
Chr9	5	26,742,297	Chr9	6	26,944,292	Chr9	3	26,964,064
Chr10	13	22,933,127	Chr10	7	21,784,612	Chr10	3	22,570,375
Chr11	14	22,193,879	Chr11	7	22,583,668	Chr11	2	22,126,735
Chr12	8	22,338,721	Chr12	2	22,219,811	Chr12	4	22,573,227
Chr13	4	21,714,992	Chr13	8	20,392,254	Chr13	8	20,728,777
Chr14	4	20,320,464	Chr14	15	22,228,334	Chr14	5	20,623,713
Chr15	7	18,227,446	Chr15	2	18,242,587	Chr15	3	18,828,828
Chr16	3	16,053,328	Chr16	3	15,238,540	Chr16	2	16,180,033
Chr17	4	15,319,648	Chr17	3	12,863,583	Chr17	3	15,471,572
Chr18	10	13,333,155	Chr18	3	16,035,322	Chr18	4	13,210,102
Chr19	5	12,198,306	Chr19	2	12,149,437	Chr19	3	12,177,556
Chr20	11	12,091,001	Chr20	3	11,859,130	Chr20	2	11,839,098
Chr21	2	8,553,409	Chr21	25	16,748,333	Chr21	10	11,589,787
Chr22	22	16,159,063	Chr22	2	8,518,109	Chr22	2	8,864,379
Chr23	7	7,977,799	Chr23	2	7,681,837	Chr23	4	7,639,852
Chr24	4	7,737,077	Chr24	3	7,641,474	Chr24	3	7,916,403
Chr25	9	7,574,820	Chr25	15	7,111,539	Chr25	17	7,193,151
Chr26	6	6,918,023	Chr26	3	6,838,665	Chr26	4	7,109,117
Chr27	18	6,270,716	Chr27	6	5,863,159	Chr27	2	6,721,840
Chr28	7	5,960,150	Chr28	8	5,811,623	Chr28	4	6,277,750
Chr29	13	1,456,683	Chr29	8	3,087,585	Chr29	2	5,803,151
Chr30	4	1,872,559	Chr30	6	2,731,120	Chr30	12	5,608,518
Chr31	7	2,637,124	Chr31	9	2,285,856	Chr31	10	4,866,990
Chr32	12	3,473,473	Chr32	3	1,658,740	Chr32	3	4,872,144
Chr33	31	2,151,773	Chr33	36	5,032,420	Chr33	9	3,839,537
Chr34	68	7,214,884	Chr34	60	6,023,616	Chr34	8	4,200,160
Chr35	58	5,548,591	Chr35	43	5,132,513	Chr35	5	3,036,682
Chr36	16	3,997,005	Chr36	14	4,162,150	Chr36	11	4,116,041
Chr37	16	3,148,754	Chr37	5	1,773,560	Chr37	2	2,878,240
Chr38	22	2,836,164	Chr38	18	1,253,703	Chr38	3	2,155,827
Chr39	11	2,018,729	Chr39	14	1,455,631	Chr39	3	1,592,768
Chr40	22	1,354,177	ChrZ	97	77,384,397	ChrZ	67	81,414,806
ChrZ	98	81,227,236						

*The pseudo-chromosomes

Supplementary Table 7. Characterization of genes in three duck genome assemblies.

	Mallard(CAU_wild_1.0)	Pekin Duck(CAU_duck_2.0)	Shaoxing Duck(CAU_laying_1.0)
Gene number	18,481	18,507	18,723
Total gene Length (bp)	440,796,430	440,512,682	441,590,005
Average gene length	23,851.33	23,802.49	23,585.43
Total exon length (bp)	2,943,099	3,012,014	3,052,411
Average exon length	159.25	162.75	163.03
Total CDS length (bp)	26,759,379	27,671,666	27,859,824
Average CDS length	1,447.94	1,495.20	1,488.00
Average exons per gene	9.09	9.19	9.13
Average intron length(bp)	2,768.52	2,724.63	2,719.62

Supplementary Table 8. Summary of gene annotation using different databases.

	Mallard(CAU_wild_1.0)		Pekin Duck(CAU_duck_2.0)		Shaoxing Duck(CAU_laying_1.0)	
	**Gene number**	**Percentage%**	**Gene number**	**Percentage%**	**Gene number**	**Percentage%**
GO	12,155	65.8	12,004	64.9	12,193	65.1
KEGG	15,160	82	15,175	82	15,383	82.2
InterPro	16,872	91.3	16,761	90.6	16,966	90.6
Pfam	14,818	80.2	14,698	79.4	14,901	79.6
Swissprot	16,976	91.9	16,934	91.5	17,139	91.5
NR	17,557	95	17,529	94.7	17,754	94.8
All_annotated	17570	95.1	17,542	94.8	17,781	95
Unannotated	911	4.9	965	5.2	942	5

Supplementary Table 9. Summary of annotation of non-coding RNA in Mallard.

	Type	Copy(w*)	Average length(bp)	Total length(bp)	% of genome
miRNA		348	90.82	31,606	0.002592
tRNA		401	74.82	30,004	0.002461
rRNA	rRNA	337	248.35	83,695	0.006864
	18S	28	721	20,188	0.001656
	28S	113	357.65	40,414	0.003315
	5.8S	9	155	1,395	0.000114
	5S	187	116.03	21,698	0.00178
snRNA	snRNA	311	125.95	39,170	0.003212
	CD-box	125	91.71	11,464	0.00094
	HACA-box	82	144.23	11,827	0.00097
	splicing	85	147.18	12,510	0.001026
	scaRNA	18	183.89	3,310	0.000271
	Unknown	1	59	59	0.000005

*The annotation of non-coding RNA was perormed using Repeamasker pipeline

Supplementary Table 10. Summary of annotation of non-coding RNA in Shaoxing duck.

	Type	Copy(w*)	Average length(bp)	Total length(bp)	% of genome
miRNA		360	91.54	32,956	0.002702
tRNA		448	74.8	33,511	0.002748
rRNA	rRNA	188	218.57	41,091	0.003369
	18S	21	710.19	14,914	0.001223
	28S	63	270.73	17,056	0.001399
	5.8S	2	155.5	311	0.000026
	5S	102	86.37	8,810	0.000722
snRNA	snRNA	327	128.31	41,958	0.00344
	CD-box	126	92.83	11,697	0.000959
	HACA-box	81	142.96	11,580	0.00095
	splicing	99	151.86	15,034	0.001233

*The annotation of non-coding RNA was perormed using Repeamasker pipeline

Supplementary Table 11. Summary of annotation of non-coding RNA in Pekin duck.

	Type	Copy(w*)	Average length(bp)	Total length(bp)	% of genome
miRNA		338	90.86	30,712	0.002574
tRNA		412	75.01	30,904	0.00259
	rRNA	133	328.22	43,653	0.003659
	18S	15	639.73	9,596	0.000804
rRNA	28S	91	337.37	30,701	0.002573
	5.8S	7	154.86	1,084	0.000091
	5S	20	113.6	2,272	0.00019
	snRNA	319	126.39	40,319	0.00338
	CD-box	128	93.66	11,989	0.001005
snRNA	HACA-box	82	142.04	11,647	0.000976
	splicing	92	148.65	13,676	0.001146
	scaRNA	16	184.25	2,948	0.000247
	Unknown	1	59	59	0.000005

*The annotation of non-coding RNA was perormed using Repeamasker pipeline

Supplementary Table 12. Summary of annotation of repeat regions.

	Mallard		Shaoxing Duck		Pekin Duck	
	Length(bp)	% in Genome	Length(bp)	% in Genome	Length(bp)	% in Genome
DNA	1,309,306	0.11	992,970	0.08	1,250,005	0.1
LINE	68,692,416	5.63	60,526,830	4.96	65,221,565	5.47
SINE	143,184	0.01	161,648	0.01	180,268	0.02
LTR	150,722,181	12.36	160,824,230	13.19	143,741,464	12.05
Unknown	10,766,233	0.88	12,037,615	0.99	12,024,687	1.01
Total	203,512,630	16.69	211,786,645	17.37	194,819,040	16.33

Supplementary Table 13. Comparison of duck genome assemblies.

	CAU_duck_1.0	BGI_duck_1.0	CAU_wild_1.0	CAU_pekin_2.0	CAU_laying_2.0
Scaffold N50 (Mb)	10.54	1.2	78	76.28	76.92
Max length of Scaffold（Mb）	52.13	5.99	208.33	207.24	212.53
Num of Super scaffold	29	47	41	40	40
Total size of Super Scaffold（Gb）	1.084	0.289	1.15	1.14	1.16
Super Scaffold（%）	91.22	26.27	94.62	95.52	95.26
Num of Gap	20679	148961	391	273	559
Total size （Gb）	1.14	1.1	1.22	1.19	1.22

Supplementary Table 14. Comparison among duck genome annotations.

Type	BGI_duck_1.0	CAU_wild_1.0
Base Pairs	1069956150	1211992756
Golden Path Length	1105035747	1211992756
Total No of Genes	16450	18490
Coding genes	15634	16765
Non coding genes	567	1270
Long non-coding RNA genes	0	789
Small non coding genes	543	476
Misc non coding genes	24	5
Pseudogenes	249	455
Total No of Transcripts	17169	29320
Protein-coding transcripts	16353	27233
miRNA transcripts	311	124
snoRNA transcripts	173	202
misc_RNA transcripts	24	5
snRNA transcripts	46	96
Genscan gene predictions	42822	48492
CDS	16353	27233
5'UTR	2450	20991
3'UTR	5983	14884
start_codon	4963	22874
stop_codon	7680	26790
exon	167967	352746

Supplementary Table 15: The "missing genes" identified in the Mallard genome

Gene	Ensembl ID	Chromosome
ZBTB39	ENSAPLG00020010241	31
GPR182	ENSAPLG00020010249	31
OLFM2	ENSAPLG00020014775	32
HIPK4	ENSAPLG00020001678	4
HSPA12B	ENSAPLG00020006121	4
WNT1	ENSAPLG00020014086	31
ADCY6	ENSAPLG00020014032	31
HOXC6	ENSAPLG00020007872	31
GPAA1	ENSAPLG00020008635	2
KLHL33	ENSAPLG00020012971	28
GABBR1	ENSAPLG00020002648	VSDN01000978.1
SYT3	ENSAPLG00020010303	VSDN01000767.1
NR4A1	ENSAPLG00020013965	31
SOX12	ENSAPLG00020006063	16
SLC44A2	ENSAPLG00020003187	VSDN01001394.1
USP39	ENSAPLG00020004963	27
GUCY2D	ENSAPLG00020001602	1
FMNL3	ENSAPLG00020013967	31
PRPH	ENSAPLG00020003492	31
SHMT2	ENSAPLG00020009673	31
CCDC65	ENSAPLG00020014077	31
CACNB3	ENSAPLG00020014058	31
DCC^	ENSAPLG00020015367	Z
KANSL2	ENSAPLG00020014022	31
MPZ	ENSAPLG00020010473	32
OPLAH	ENSAPLG00020008651	2
SGCA	ENSAPLG00020002120	25
ILF3	ENSAPLG00020015171	VSDN01000121.1
CYP27B1	ENSAPLG00020014157	31
OSBPL7	ENSAPLG00020004951	25
FARSA*	ENSAPLG00020007675	27
TBX21	ENSAPLG00020005095	25
ERBB3	ENSAPLG00020013649	31
TBKBP1^	ENSAPLG00020005117	25
ALDOA	ENSAPLG00020003661	VSDN01000051.1
PPP5C	ENSAPLG00020012123	VSDN01001265.1
MYL6	ENSAPLG00020013461	31
POLI	ENSAPLG00020015354	Z
TARBP2	ENSAPLG00020008020	31
COL5A3*	ENSAPLG00020014778	32
RAB27B	ENSAPLG00020015337	Z
STAT6^	ENSAPLG00020010194	31
SLC6A8†	ENSAPLG00020015407	10
RAB5B	ENSAPLG00020013656	31
PDLIM2	ENSAPLG00020004203	27
ATG4D	ENSAPLG00020004087	VSDN01001394.1
PACS1†	ENSAPLG00020008632	3
JOSD2	ENSAPLG00020007700	37
BLVRB	ENSAPLG00020003358	34
RCE1	ENSAPLG00020004218	VSDN01001083.1
YIF1B^	ENSAPLG00020012130	VSDN01001265.1
ANKRD39	ENSAPLG00020002413	27
SAE1	ENSAPLG00020003419	34
ESYT1^	ENSAPLG00020013465	31
FLOT1	ENSAPLG00020002758	VSDN01000978.1
KRI1	ENSAPLG00020004064	VSDN01001394.1
LETMD1	ENSAPLG00020014329	31
METTL1	ENSAPLG00020014159	31
TSFM	ENSAPLG00020014165	31
RPS26	ENSAPLG00020013650	31
STAC3	ENSAPLG00020009658	31
PPOX	ENSAPLG00020010528	32
TMEM150A†	ENSAPLG00020013628	7
ARHGAP9	ENSAPLG00020009570	31
UBA1	ENSAPLG00020018435	10
TINF2	ENSAPLG00020003027	40
TECR	ENSAPLG00020011341	8
B9D2*	ENSAPLG00020003062	34
DDIT3	ENSAPLG00020009593	31
FKBP11	ENSAPLG00020014080	31
TMEM88†	ENSAPLG00020018119	21
RBCK1†	ENSAPLG00020008590	2
DAZAP2	ENSAPLG00020014348	31
DCTN2	ENSAPLG00020002106	16
UBL5	ENSAPLG00020003852	32
TMEM147	ENSAPLG00020011569	VSDN01000824.1
TMEM205†	ENSAPLG00020016383	8
STAP2	ENSAPLG00020012553	28
STARD6	ENSAPLG00020015349	Z
RLN3†	ENSAPLG00020009436	Z
IRF3†	ENSAPLG00020017677	5
RABGGTA	ENSAPLG00020002580	40
CCDC68	ENSAPLG00020015333	Z
PDZD4	ENSAPLG00020004953	1
C12ORF44	ENSAPLG00020013966	31
C18ORF54	ENSAPLG00020015345	Z
C1ORF192	ENSAPLG00020010461	32
C2ORF68	ENSAPLG00020005100	27
CCDC88B†	ENSAPLG00020011425	3

† The `missing genes` only annotated in the wild duck genome, but missing annotations in chicken genomes.
* The `missing genes` were annotated to lncRNA in mallard genome.
^ The `missing genes` were annotated to pseudogene genes in mallard genome.

In the annotated transcripts of the wild duck genome, we found 89 genes that have been thought to be missing in chickens now[22].

Supplementary Table 16: GC content of annotated missing genes in Mallard and human.

Gene	Ensembl ID	Mallard(%)	Human(%)
B9D2	ENSAPLG00020003062	59.84	62.22
FARSA	ENSAPLG00020007675	66.40	61.29
COL5A3	ENSAPLG00020014778	68.76	64.22
TBKBP1	ENSAPLG00020005117	70.05	61.80
STAT6	ENSAPLG00020010194	63.61	56.09
YIF1B	ENSAPLG00020012130	67.51	63.93
ESYT1	ENSAPLG00020013465	71.72	58.15
DCC	ENSAPLG00020015367	58.03	43.24
GUCY2D	ENSAPLG00020001602	50.44	65.16
HIPK4	ENSAPLG00020001678	58.33	62.80
DCTN2	ENSAPLG00020002106	56.57	51.55
SGCA	ENSAPLG00020002120	68.74	62.81
ANKRD39	ENSAPLG00020002413	72.14	56.21
RABGGTA	ENSAPLG00020002580	74.06	60.00
GABBR1	ENSAPLG00020002648	63.34	54.34
FLOT1	ENSAPLG00020002758	71.20	54.14
TINF2	ENSAPLG00020003027	72.60	44.02
SLC44A2	ENSAPLG00020003187	58.15	57.34
BLVRB	ENSAPLG00020003358	71.20	63.33
SAE1	ENSAPLG00020003419	61.77	49.93
PRPH	ENSAPLG00020003492	71.74	59.75
ALDOA	ENSAPLG00020003661	68.00	60.96
UBL5	ENSAPLG00020003852	70.44	50.61
KRI1	ENSAPLG00020004064	68.95	57.48
ATG4D	ENSAPLG00020004087	69.80	64.95
PDLIM2	ENSAPLG00020004203	70.44	66.45
RCE1	ENSAPLG00020004218	71.00	63.15
OSBPL7	ENSAPLG00020004951	71.97	61.03
PDZD4	ENSAPLG00020004953	46.27	64.27
USP39	ENSAPLG00020004963	67.86	53.86
TBX21	ENSAPLG00020005095	72.25	59.62
C2ORF68	ENSAPLG00020005100	80.59	47.29
SOX12	ENSAPLG00020006063	70.05	61.99
HSPA12B	ENSAPLG00020006121	68.85	64.51
JOSD2	ENSAPLG00020007700	62.52	68.08
HOXC6	ENSAPLG00020007872	49.07	56.32
TARBP2	ENSAPLG00020008020	72.42	61.02
RBCK1	ENSAPLG00020008590	72.11	59.70
PACS1	ENSAPLG00020008632	65.32	59.48
GPAA1	ENSAPLG00020008635	70.11	66.17
OPLAH	ENSAPLG00020008651	73.58	66.57
RLN3	ENSAPLG00020009436	55.72	59.96
ARHGAP9	ENSAPLG00020009570	69.10	58.58
DDIT3	ENSAPLG00020009593	66.45	50.80
STAC3	ENSAPLG00020009658	62.81	55.95
SHMT2	ENSAPLG00020009673	65.44	56.14
ZBTB39	ENSAPLG00020010241	61.15	49.90
GPR182	ENSAPLG00020010249	57.44	54.47
SYT3	ENSAPLG00020010303	68.55	57.32
C1ORF192	ENSAPLG00020010461	71.98	47.80
MPZ	ENSAPLG00020010473	69.22	55.11
PPOX	ENSAPLG00020010528	73.38	63.01
TECR	ENSAPLG00020011341	43.65	58.06
CCDC88B	ENSAPLG00020011425	40.57	67.41
TMEM147	ENSAPLG00020011569	73.50	56.91
PPP5C	ENSAPLG00020012123	61.98	58.58
STAP2	ENSAPLG00020012553	70.51	59.66
KLHL33	ENSAPLG00020012971	61.13	52.43
MYL6	ENSAPLG00020013461	60.94	53.39
TMEM150A	ENSAPLG00020013628	68.53	58.56
ERBB3	ENSAPLG00020013649	66.83	51.05
RPS26	ENSAPLG00020013650	64.59	47.96
RAB5B	ENSAPLG00020013656	63.12	48.31
NR4A1	ENSAPLG00020013965	68.55	62.04
C12ORF44	ENSAPLG00020013966	68.62	59.68
FMNL3	ENSAPLG00020013967	65.45	51.99
KANSL2	ENSAPLG00020014022	65.94	44.12
ADCY6	ENSAPLG00020014032	67.79	54.25
CACNB3	ENSAPLG00020014058	66.73	51.99
CCDC65	ENSAPLG00020014077	68.26	46.59
FKBP11	ENSAPLG00020014080	74.26	51.54
WNT1	ENSAPLG00020014086	68.23	63.21
CYP27B1	ENSAPLG00020014157	72.09	56.32
METTL1	ENSAPLG00020014159	71.45	53.12
TSFM	ENSAPLG00020014165	75.48	44.91
LETMD1	ENSAPLG00020014329	74.42	49.75
DAZAP2	ENSAPLG00020014348	71.30	43.99
OLFM2	ENSAPLG00020014775	63.28	61.32
ILF3	ENSAPLG00020015171	62.38	55.77
CCDC68	ENSAPLG00020015333	59.44	35.74
RAB27B	ENSAPLG00020015337	57.97	46.30
C18ORF54	ENSAPLG00020015345	45.07	38.27
STARD6	ENSAPLG00020015349	43.76	40.70
POLI	ENSAPLG00020015354	48.10	36.77
SLC6A8	ENSAPLG00020015407	68.09	62.96
TMEM205	ENSAPLG00020016383	69.31	58.52
IRF3	ENSAPLG00020017677	61.46	60.81
TMEM88	ENSAPLG00020018119	56.04	61.76
UBA1	ENSAPLG00020018435	68.40	56.35

Supplementary Table 17. Summary of putative presence/absence variations in duck genomes.

Term	PAV Num	Total Length(Mb)
Pekin duck v.s. Mallard	6138	6.95
Shaoxing duck v.s. Mallard	7710	8.14
Mallard v.s. Pekin duck	6216	7.55
Mallard v.s. Shaoxing duck	7810	12.03

Supplementary Table 18. Representative peaks of ATAC-Seq for differentiated adipocytes at Chr 12: 17.213 – 17.218 Mb.

Chr	Start	End	Tissue	Strand	Peak Score	Detailed Annotation	Distance to TSS(bp)	Entrez ID
Chr12	17213000	17216790	Abdominal fat	+	355	rotein_coding-intron (ENSAPLT00020021865, intron 1 of [illegible]	1205	ENSAPLG00020014265
Chr12	17215280	17217880	Abdominal fat	+	3122	rotein_coding-intron (ENSAPLT00020021865, intron 1 of [illegible]	-2398	ENSAPLG00020014265
Chr12	17213460	17214420	Abdominal fat	+	75	rotein_coding-intron (ENSAPLT00020021865, intron 1 of [illegible]	250	ENSAPLG00020014265
Chr12	17214270	17216790	Abdominal fat	+	343	rotein_coding-intron (ENSAPLT00020021865, intron 1 of [illegible]	1840	ENSAPLG00020014265
Chr12	17215800	17217880	Abdominal fat	+	1735	rotein_coding-intron (ENSAPLT00020021865, intron 1 of [illegible]	-2138	ENSAPLG00020014265
Chr12	17213400	17216630	Abdominal fat	+	322	rotein_coding-intron (ENSAPLT00020021865, intron 1 of [illegible]	1325	ENSAPLG00020014265
Chr12	17215220	17217880	Abdominal fat	+	3025	rotein_coding-intron (ENSAPLT00020021865, intron 1 of [illegible]	-2428	ENSAPLG00020014265
Chr12	17213380	17216780	Abdominal fat	+	278	rotein_coding-intron (ENSAPLT00020021865, intron 1 of [illegible]	1390	ENSAPLG00020014265
Chr12	17215310	17217880	Abdominal fat	+	2887	rotein_coding-intron (ENSAPLT00020021865, intron 1 of [illegible]	-2383	ENSAPLG00020014265
Chr12	17213240	17214450	Subcutaneous fat	+	92	rotein_coding-intron (ENSAPLT00020021865, intron 1 of [illegible]	155	ENSAPLG00020014265
Chr12	17214620	17216630	Subcutaneous fat	+	277	rotein_coding-intron (ENSAPLT00020021865, intron 1 of [illegible]	1935	ENSAPLG00020014265
Chr12	17215510	17217880	Subcutaneous fat	+	2987	rotein_coding-intron (ENSAPLT00020021865, intron 1 of [illegible]	-2283	ENSAPLG00020014265
Chr12	17213570	17214430	Subcutaneous fat	+	126	rotein_coding-intron (ENSAPLT00020021865, intron 1 of [illegible]	310	ENSAPLG00020014265
Chr12	17214230	17215750	Subcutaneous fat	+	321	rotein_coding-intron (ENSAPLT00020021865, intron 1 of [illegible]	1300	ENSAPLG00020014265
Chr12	17216430	17217880	Subcutaneous fat	+	3214	rotein_coding-intron (ENSAPLT00020021865, intron 1 of [illegible]	-1823	ENSAPLG00020014265
Chr12	17213390	17216630	Subcutaneous fat	+	458	rotein_coding-intron (ENSAPLT00020021865, intron 1 of [illegible]	1320	ENSAPLG00020014265
Chr12	17215750	17217880	Subcutaneous fat	+	3309	rotein_coding-intron (ENSAPLT00020021865, intron 1 of [illegible]	-2163	ENSAPLG00020014265
Chr12	17213380	17214440	Subcutaneous fat	+	98	rotein_coding-intron (ENSAPLT00020021865, intron 1 of [illegible]	220	ENSAPLG00020014265
Chr12	17214200	17216250	Subcutaneous fat	+	458	rotein_coding-intron (ENSAPLT00020021865, intron 1 of [illegible]	1535	ENSAPLG00020014265
Chr12	17216400	17217880	Subcutaneous fat	+	2945	rotein_coding-intron (ENSAPLT00020021865, intron 1 of [illegible]	-1838	ENSAPLG00020014265

Supplementary Table 19. Primer sequences used for the PCR, Q-PCR, and RACE analysis.

Species	Gene	Primer for PCR	Tm(℃)	Length(bp)
Pekin Duck	ACA-1	Forward：5'-GACACAGGAAGGACGAGGATG-3'	60.1	645
		Reverse：5'-CTTCGTGCGCATTTATTGAGGG-3'	60.5	
	ACA-2	Forward：5'-CCAAGTGCGTCGGTGAGG-3'	60.7	720
		Reverse：5'-CGCTGCTTCGCTCTAGGA-3'	59.2	
Sichuan goose	ANCA-1	Forward：5'-CTCTGCGTGCTGGGATTCATGGC-3'	62.9	451
		Reverse：5'-GCTAGGCTGTGTATTTGAAGGCGAAGG-3'	62.6	
	ANCA-2	Forward：5'-CTTCGGCTCCAGGACCCA-3'	61	343
		Reverse：5'-CAGTGTGGGCGCTGTGTC-3'	61.3	
King pigeon	CCA-1	Forward：5'-CCACAGGAAGGACGAGGAT-3'	60.1	667
		Reverse：5'-TGCTTCGTGTGCGTTTATTG-3'	60.8	
	CCA-2	Forward：5'-CCAGAAGCAGGGTGCTGA-3'	61.1	683
		Reverse：5'-GAGATCAGGGCCTTTATTGG-3'	58.6	
	CCA-3	Forward：5'-TGGGAACTCAGCAGAAGCCGTTATTC-3'	61.9	681
		Reverse：5'-AGAGGAGTAGAGGTGCCAAGGAAGGA-3'	62.8	
Japanese quail	COCA	Forward：5'-AGCTGCGCAAACAGTGACTCCGGTG-3'	69.7	842
		Reverse：5'-CTCATCACGCACCACCGTCCCTCCT-3'	70	
Zebra finch	TCA	Forward 2：5'-TGGGACGAGGCTGAGAGTTGGT-3'	61.3	127
		Reverse 2：5'-AGATGGGCTGCACACGCTGGTA-3'	62.5	

Species	Gene	primer for RACE		
Sichuan Goose	ANCA-1	5'-GSP：5'-GATTACGCCAAGCTTGCTGGAACTTGGCGACGAACCT-3'	68.1	365
		3'-GSP：5'-GATTACGCCAAGCTTTGGAGGAAGGCAGAGGCTTGGT-3'	68.3	596
	ANCA-2	5'-GSP：5'-GATTACGCCAAGCTTCAGTGTGGGCGCTGTGTC-3'	67.5	700
Zebra Finch	TCA	5'-GSP：5'-GATTACGCCAAGCTTAGATGGGCTGCACACGCTGGTA-3'	68.7	400
		3'-GSP：5'-GATTACGCCAAGCTTTGGGACGAGGCTGAGAGTTGGT-3'	69.4	533
