# Supplementary material for: Three chromosome-level duck genome assemblies provide insights into genomic variation during domestication
Source: Nat Commun. 2021 Oct 11;12:5932. doi: 10.1038/s41467-021-26272-1 (PMC8505442; doi:10.1038/s41467-021-26272-1)
Supplement: Supplementary file 2 — Description of Additional Supplementary Files [file 41467_2021_26272_MOESM2_ESM.pdf]

Description of Additional Supplementary Files

File Name: Supplementary Data 1
Description: The summary of radiation hybrid-map (RH) markers mapping to duck genomes.

File Name: Supplementary Data 2
Description: Mammalian or lizard genes that are not present in mallard transcripts.

File Name: Supplementary Data 3
Description: The transcripts of the paralogs of the `missing genes` in the Mallard genome.

File Name: Supplementary Data 4
Description: Annotation of putative PAVs in duck genomes.

File Name: Supplementary Data 5
Description: Information on validated PAVs in duck genomes.

File Name: Supplementary Data 6
Description: Enrichment analysis for validated PAVs.

File Name: Supplementary Data 7
Description: Differentially expressed genes between Pekin duck and Mallard in different tissues.

File Name: Supplementary Data 8
Description: Information on putative SVs in duck genomes.

File Name: Supplementary Data 9
Description: The SNP frequency within gene regions (including upstream and downstream regions).

File Name: Supplementary Data 10
Description: Details for sample sequencing.

File Name: Supplementary Data 11
Description: Sequence information for the CTL family used in phylogenetic analysis.
