# Supplementary material for: Three chromosome-level duck genome assemblies provide insights into genomic variation during domestication
Source: Nat Commun. 2021 Oct 11;12:5932. doi: 10.1038/s41467-021-26272-1 (PMC8505442; doi:10.1038/s41467-021-26272-1)
Supplement: Supplementary file 14 — Reporting Summary [file 41467_2021_26272_MOESM14_ESM.pdf]

nature research

Corresponding author(s): Zhuo-Cheng Hou
Last updated by author(s): Jul 30, 2021

Reporting Summary

Nature Research wishes to improve the reproducibility of the work that we publish. This form provides structure for consistency and transparency in reporting. For further information on Nature Research policies, see our Editorial Policies and the Editorial Policy Checklist.

Statistics

For all statistical analyses, confirm that the following items are present in the figure legend, table legend, main text, or Methods section.

n/a	Confirmed	
☐	☒	The exact sample size (n) for each experimental group/condition, given as a discrete number and unit of measurement
☐	☒	A statement on whether measurements were taken from distinct samples or whether the same sample was measured repeatedly
☐	☒	The statistical test(s) used AND whether they are one- or two-sided<br>*Only common tests should be described solely by name; describe more complex techniques in the Methods section.*
☒	☐	A description of all covariates tested
☒	☐	A description of any assumptions or corrections, such as tests of normality and adjustment for multiple comparisons
☐	☒	A full description of the statistical parameters including central tendency (e.g. means) or other basic estimates (e.g. regression coefficient) AND variation (e.g. standard deviation) or associated estimates of uncertainty (e.g. confidence intervals)
☐	☒	For null hypothesis testing, the test statistic (e.g. F, t, r) with confidence intervals, effect sizes, degrees of freedom and P value noted<br>*Give P values as exact values whenever suitable.*
☒	☐	For Bayesian analysis, information on the choice of priors and Markov chain Monte Carlo settings
☒	☐	For hierarchical and complex designs, identification of the appropriate level for tests and full reporting of outcomes
☐	☒	Estimates of effect sizes (e.g. Cohen's d, Pearson's r), indicating how they were calculated

Our web collection on statistics for biologists contains articles on many of the points above.

Software and code

Policy information about availability of computer code

Data collection	Falcon v0.3, Quiver v2.3.0, Pilon v1.20, Augustus v2.5.5, IrysView v2.5.1 ,PBSuite v14.9.9, Lachesis(Jan 2017), LR_Gapcloser(Sep 2018), LastZ, BLAST v2.5.0, Burrows-Wheeler Aligner v0.7.10-r789, Bowtie2 v2.3.5.1, SAMtools v0.1.19, Trimmomatic v0.32, Genewise v2.4.1, GlimmerHMM(v3.0.4),AutoDedect(v2.1.4) ,SNAP (4 Jun 2019),Geneid (v 1.4.4), Genscan(Feb. 18, 2003), Pasa v2.3.1, Cufflink v2.2.1, EVidenceModeler v1.1.1, Tandem Repeats Finder v4.09, LTR_FINDER v1.06, RepeatModeler v1.05 and RepeatMasker v4.0.6
Data analysis	HISAT2 v2.1.0, Primer 3 v0.4.0,HTSeq v0.9.1, MCScanX(2014-06-10), BEDTools v2.27.1, vcflibs v1.0, Metascape (2019-8-14), Homer v4.11, BUSCO (version 2), ESPript (version 3.0) , Speedseq v0.1.2, MUMmer v4.00beta2, MEGA (version 10), Variant Effect Predictor (release 100), HMMRATAC v1.2.2 and DESeq2 package v.1.24.0 in R version 3.5.2

For manuscripts utilizing custom algorithms or software that are central to the research but not yet described in published literature, software must be made available to editors and reviewers. We strongly encourage code deposition in a community repository (e.g. GitHub). See the Nature Research guidelines for submitting code & software for further information.

Data

Policy information about availability of data

All manuscripts must include a data availability statement. This statement should provide the following information, where applicable:
- Accession codes, unique identifiers, or web links for publicly available datasets
- A list of figures that have associated raw data
- A description of any restrictions on data availability

All genome assembly datasets reported in this study have been deposited in GenBank (NCBI) and BIG with the following accession codes: Mallard genome, PRJNA554956; Pekin duck genome, (GenBank, JACEUL000000000; BIG, GWHANUR00000000); Shaoxing duck genome (GenBank, JACEUM000000000; BIG, GWHANUS00000000). All datasets have also been deposited in the Genome Warehouse of the BIG Data Center at the Beijing Institute of Genomics, Chinese

Academy of Sciences, under the following accession numbers: whole-genome re-sequencing data, CRA002746; whole-genome sequencing data of Shaoxing duck, CRA002750 and CRA002733; dynamic transcriptome sequencing of Mallard liver tissue, CRA002743; dynamic transcriptome sequencing of Mallard skin fat tissue, CRA002755; dynamic transcriptome sequencing of Pekin duck liver tissue, CRA002747; dynamic transcriptome sequencing of Pekin duck skin fat tissue, CRA002754; RNA-Seq of Mallard subcutaneous preadipocytes, CRA002775. The RNA-Seq of Pekin duck subcutaneous preadipocytes92, the Iso-Seq of Pekin duck33, and whole-genome re-sequencing of Mallard1 have been reported previously, and the data were deposited into the NCBI database under accession numbers SRX4646736, SRP188279, PRJNA450892, respectively. The raw sequencing data also reported in this paper have been deposited in the Sequence Read Archive (SRA) under NCBI BioProject accession PRJNA645648 and PRJNA554956. The sequence source of the public database were shown below: Uniprot database was downloaded from https://www.uniprot.org/; Ensembl/GENCODE gene set of human was downloaded from http://ftp.ensembl.org/pub/release-103/fasta/homo_sapiens/pep/Homo_sapiens.GRCh38.pep.all.fa.gz; Nr database was downloaded from https://ftp.ncbi.nlm.nih.gov/blast/db/FASTA/nr.gz; KEGG database was downloaded from https://www.genome.jp/kegg/; InterPro was downloaded from https://www.ebi.ac.uk/interpro/; Pfam database was downloaded from http://pfam.xfam.org/; GO database was downloaded from http://geneontology.org/. All data and research materials are available upon reasonable request by contacting the corresponding authors.

Field-specific reporting

Please select the one below that is the best fit for your research. If you are not sure, read the appropriate sections before making your selection.

☒ Life sciences ☐ Behavioural & social sciences ☐ Ecological, evolutionary & environmental sciences

For a reference copy of the document with all sections, see nature.com/documents/nr-reporting-summary-flat.pdf

Life sciences study design

All studies must disclose on these points even when the disclosure is negative.

Sample size

No sample-size calculation was performed,but the sample size chosen was compare to the pervious literatures similarly reporting genomic resources for domesticated and wild species. For instance ,Zhou et al.2018 used 40 mallard and 1000 domestic duck for exploring genetic variation and genetic mechanism of domestication and selection. Therefore, we think in our study ,the samples size is sufficient for genomic and transcriptomic analysis. Additionally,we chose the sample size of individual animal and cell molecular biology experiments based on past experience on detecting differences with a given method as previously applied in (Pei Liu et al. Nature communication, 2019; Diana Teh Chee Siang et. al. Nature communication, 2020; Lisa Suwandhi et al. Nature communication, 2021). We and the above researchers both use at least three samples for tests in a single experiment.

Data exclusions

Data was only excluded where experiments failed (based on animals or cells failed to meet experiment endpoint or variance of internal standards), or where limited material were exhausted.

Replication

Differentiation assays have been performed at least three times, and all attempt at replication were successfully (detail are indicated on figures/results). In RNA-seq analysis, each design has 6 biological replicates, which eliminates intra-group errors and improves the accuracy of the results. The sequencing data is 8G/sample to ensure that the sequencing is saturated. DNA re-sequencing sequencing depth is between 10-30x to ensure comprehensive and accurate detection of variation. We aimed to address all conclusions using different methods and replicated each experiment as written in the respective figure legends. Each experiment was repeated at least triple under independent conditions, all replicates showed similar results.

Randomization

All samples were randomly assigned to experimental groups. Animals were weight matched and randomly assigned to experimental groups. Cells in culture wells were randomly assigned to study groups.

Blinding

Blinding was not used because all analyses in the main text were performed on the premise that the samples are known.

Reporting for specific materials, systems and methods

We require information from authors about some types of materials, experimental systems and methods used in many studies. Here, indicate whether each material, system or method listed is relevant to your study. If you are not sure if a list item applies to your research, read the appropriate section before selecting a response.

Materials & experimental systems

n/a	Involved in the study	
☒	☐	Antibodies
☐	☒	Eukaryotic cell lines
☒	☐	Palaeontology and archaeology
☐	☒	Animals and other organisms
☒	☐	Human research participants
☒	☐	Clinical data
☒	☐	Dual use research of concern

Methods

n/a	Involved in the study	
☒	☐	ChIP-seq
☒	☐	Flow cytometry
☒	☐	MRI-based neuroimaging

Eukaryotic cell lines

Policy information about cell lines

Cell line source(s)	The ICP1 cell line used in this study is an immortalized chicken preadipocytes generated by transduction chicken telomerase reverse transcriptase gene, which is preserved by the Poultry Breeding Group of the College of Animal Science and Technology, Northeast Agricultural University, China.<br>Reference:<br>Wang W, Zhang T, Wu C, Wang S, Wang Y, Li H, Wang N: Immortalization of chicken preadipocytes by retroviral transduction of chicken TERT and TR. PLoS One 2017, 12(5):e0177348.
Authentication	The cells were authenticated by morphology and differentiated potency.
Mycoplasma contamination	Cells were not tested for mycoplasma contamination but such contamination would not have allowed for proper ciliation
Commonly misidentified lines<br>(See ICLAC register)	No commonly misidentified cell lines were used

Animals and other organisms

Policy information about studies involving animals; ARRIVE guidelines recommended for reporting animal research

Laboratory animals	For DNA and RNA collection,Laboratory animals were used. Pekin duck,Mallard and Shaoxing duck used for genome assembly are all adults.18 Pekin ducks and 18 Mallard aged 2, 4 and 6 weeks were used to collect RNA. Forty-eight (male: 23, female:25) 42-day Pekin ducks, 23 Shaoxing adult female ducks and 1 female adult Mallard were used for DNA collection. 6 female Pekin ducks and 6 female Mallard at age of 2 weeks were used to collect subcutaneous preadipocytes.
Wild animals	The study did not involve wild animals
Field-collected samples	The study did not involve the sample collected from the field
Ethics oversight	Animal Care and Use Committee of China Agricultural University (permit number: SYXK 2007–0023).

Note that full information on the approval of the study protocol must also be provided in the manuscript.
